# Light and protonation-controlled complex formation between sulfate ions and a stiff-stilbene based bis(cyclopeptide)†

**DOI:** 10.1039/d5sc00766f

**Published:** 2025-03-24

**Authors:** Stefan Mommer, Benedict Wyrwol, Jasper E. Bos, Stefan Kubik, Sander J. Wezenberg

**Affiliations:** a Leiden Institute of Chemistry, Leiden University Einsteinweg 55 2333 CC Leiden The Netherlands s.j.wezenberg@lic.leidenuniv.nl; b Fachbereich Chemie–Organische Chemie, RPTU Kaiserslautern-Landau Erwin-Schrödinger-Str. 54 67663 Kaiserslautern Germany stefan.kubik@rptu.de

## Abstract

Anion–ligand coordination has been used to generate a number of supramolecular structures. Of particular interest is the transformation between different types of complexes using various stimuli. While there are multiple examples where this has been achieved with metal–ligand coordination complexes through incorporation of molecular photoswitches, the same has not yet been realized with anion–ligand coordination-driven assemblies. In this study, a sulfate-binding bis(cyclopeptide) with a photoswitchable stiff-stilbene linker is presented. Its (*E*)- and (*Z*)-isomers, and the different degrees of protonation of the anion (HSO_4_^−^*vs.* SO_4_^2−^), give rise to different assembly states. The accessible products have 1 : 1, 1 : 2 and 2 : 2 host–guest stoichiometries and can be interconverted by light irradiation and acid/base addition, resulting in a highly controllable responsive system that demonstrates the potential of sulfate coordination-driven supramolecular assembly.

## Abstract 2

A sulfate-binding stiff-stilbene based bis(cyclopeptide) gives rise to 1 : 1, 1 : 2 and 2 : 2 host–guest assemblies, which can be interconverted by light irradiation and acid/base addition.

## Graphical abstract

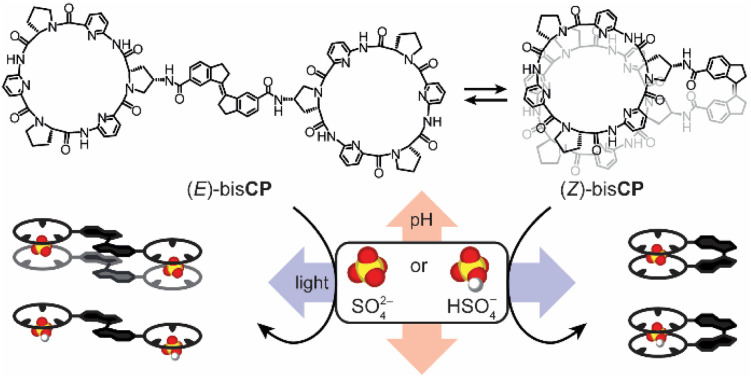

## Introduction

Coordination chemistry has been used to assemble a wide range of nanostructures, including supramolecular boxes, cages, and helicates.^1^ Recent efforts have been directed at inducing reversible transformations between different types of metal–ligand complexes, as a means to control their properties and functions.^2^ Among the stimuli that have been applied to achieve this, light has proven to be highly promising as it can be delivered with high spatiotemporal precision without the build-up of waste products.^3^ The main strategy here is to incorporate molecular photoswitches into the ligands.^4^ However, the heavy metal atoms often used to assemble these nanostructures can be toxic, limiting biological application.

Anions are ubiquitous in biological and chemical processes. In recent years, they have gradually become important motifs in coordination driven self-assembly.^5,6^ They exhibit a rich variety of geometries and coordination sites and hence, in combination with suitable hydrogen bond-donating ligands, various supramolecular architectures can be accessed.^5,6^ While these developments pave the way to a new generation of self-assembled structures and materials with new functions, the incorporation of photoswitchable units has not yet been realized.

**Scheme 1:**
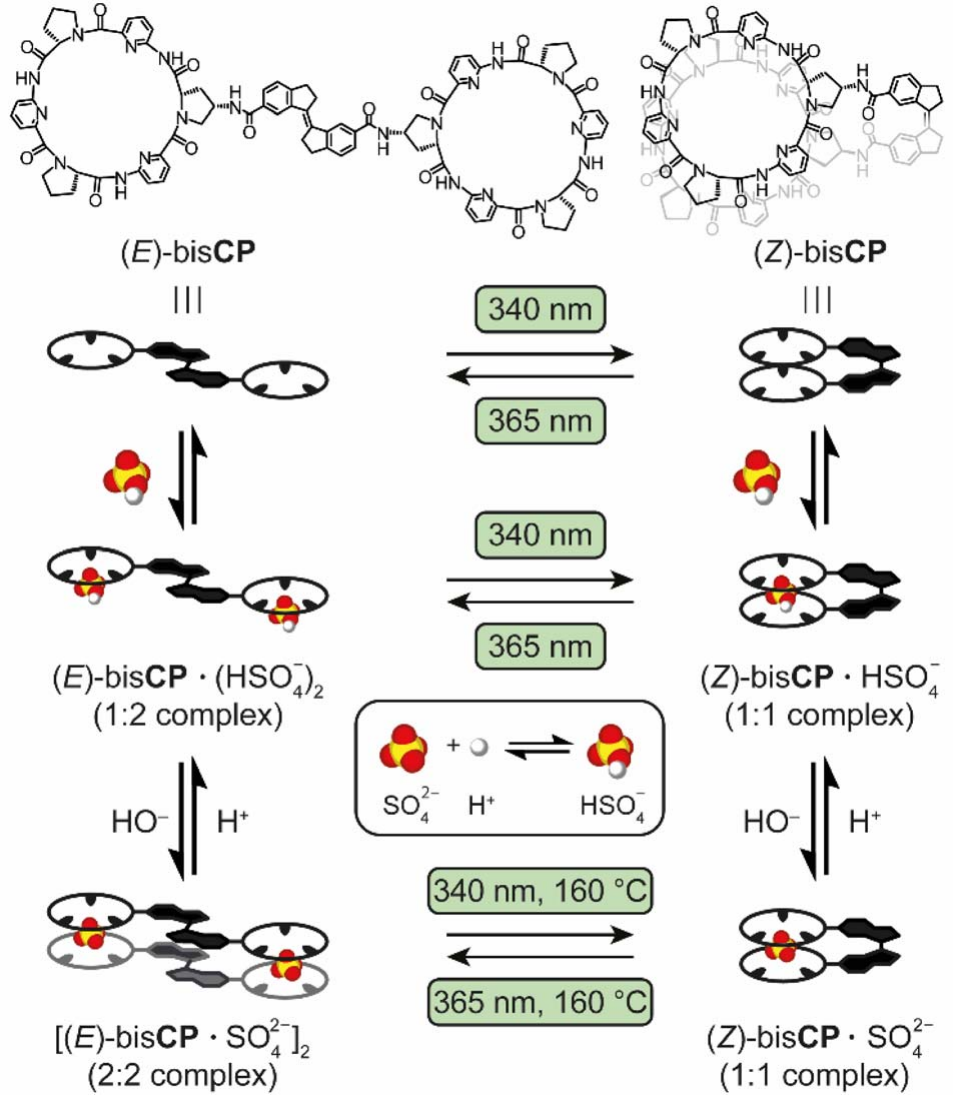
Light- and protonation-controlled switching between stiff-stilbene-bridged bisCP in different isomeric and HSO_4_^−^/SO_4_^2−^ anion-complexed states.

Inspired by these developments and motivated to gain control over anion–ligand complex formation using light, we designed the photoswitchable bis(cyclopeptides) (*E*)-bisCP and (*Z*)-bisCP containing a rigid stiff-stilbene linker (Scheme 1). Analogous bis(cyclopeptides) with flexible linkers have previously been shown to form complexes with anions such as sulfate and halides, in which the anion resides in a cavity between the two cyclopeptide rings and interacts with six converging peptide NH groups.^7^ Anion binding was studied mainly in aqueous solvent mixtures and in water, where complex formation was largely driven by the desolvation of the anion binding site and the hydrophobic proline residues when the two receptor halves came together, but studies have also been performed in acetonitrile and DMSO.^8^ In the latter solvent, iodide binding is negligible since it does not benefit from solvophobic effects, while binding to the strongly coordinating sulfate anion is substantial.

The stiff-stilbene photoswitch was used previously as a backbone of tweezer-type molecular anion receptors, which exhibited large differences in binding affinity between the (*E*)- and (*Z*)-isomers.^9^ Its use as a photoswitchable linker is advantageous owing to its large geometrical change upon isomerization and its high thermal stability.^10^ While a significant number of photo-switchable anion receptors have been developed over the past decade,^11,12^ it should be noted that the photocontrolled binding of sulfate is largely unexplored.^13^ In the present case, we anticipated that incorporation of stiff-stilbene between the cyclopeptide anion-binding moieties would afford a receptor that in the (*Z*)-form is able to bind the sulfate anion by simultaneous interaction with these moieties (see Scheme 1). In contrast, in the (*E*)-form the cyclopeptide residues would be too far apart from each other to give a 1 : 1 host–guest complex and higher-order species could therefore be formed. In addition, the use of DMSO as the solvent would allow the protonation of the sulfate dianion (SO_4_^2−^) and the deprotonation of the corresponding hydrogen sulfate (HSO_4_^−^) anion to be used as independent stimuli to control complex formation (the p*K*_a_ of HSO_4_^−^ is 2.0 in water and 14.5 in DMSO),^14^ as was previously demonstrated for another system by Chmielewski and co-workers.^15^

Here, we show that reversible photoisomerisation of the rigid stilbene linker in bisCP can be achieved by irradiation with light of different wavelengths. The (*Z*)-isomer binds HSO_4_^−^ and SO_4_^2−^ with different affinities, but 1 : 1 complexes are formed in both cases. In contrast, the (*E*)-isomer forms a 1 : 2 complex with HSO_4_^−^, while a distinct 2 : 2 sandwich-type complex is formed in the presence of dianionic SO_4_^2−^ (see Scheme 1). Thus, the mode of sulfate binding not only depends on the configuration of the linker but also on the degree of protonation of the anion. The multiple parameters controlling the supramolecular assembly make this system highly dynamic, and bisCP thus represents a unique example of a switchable receptor whose binding mode can be reversibly controlled by two orthogonal stimuli.

## Results and discussion

### Synthesis and photoswitching behaviour

The configurational isomers (*E*)- and (*Z*)-bisCP were synthesized independently by a TBTU-mediated coupling of the respective stiff-stilbene bis(carboxylic acid) with a known cyclopeptide containing one (2*S*,4*S*)-4-aminoproline subunit (Fig. S1 in the ESI†).^8,16^ The (*E*)-isomer of the stiff-stilbene bis(carboxylic acid) was prepared using an adapted protocol from Akbulatov *et al.*,^17^ while the (*Z*)-isomer was obtained using a procedure that some of us reported previously.^12*g*^ Upon completion of the amide coupling reactions, the products were precipitated and purified by preparative HPLC. They were obtained in analytically pure form in yields of 51% for (*E*)-bisCP and 32% for (*Z*)-bisCP (Fig. S2–S7 in the ESI†).

**Fig. 1:**
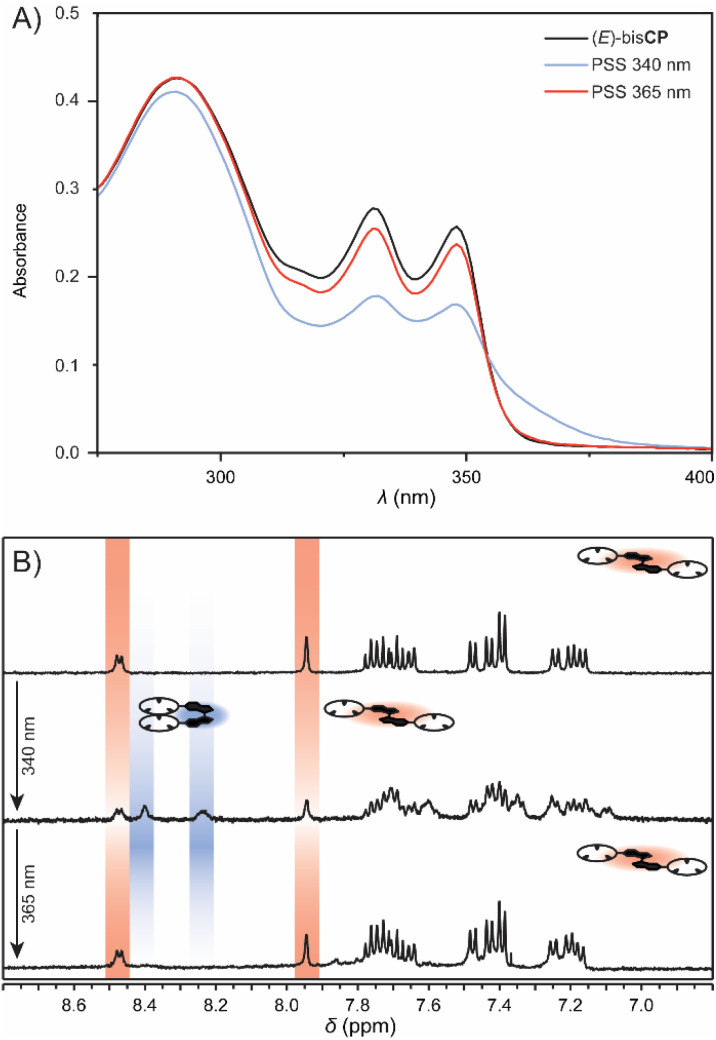
(A) UV-vis spectral changes of (*E*)-bisCP in 0.5 vol% H_2_O/DMSO (10 μM) upon irradiation at 340 and 365 nm. (B) ^1^H NMR spectra of (*E*)-bisCP in 0.5 vol% H_2_O/DMSO-*d*_6_ (0.5 mM) (top), after irradiation at 340 nm (middle) and 365 nm (bottom).

The photoswitching properties of the bis(cyclopeptides) were studied first by UV-vis spectroscopy. The absorbance spectrum of (*E*)-bisCP in 0.5 vol% H_2_O/DMSO^18^ (*c* = 10 μM) showed three maxima at *λ* = 290, 331 and 347 nm (Fig. 1A). When the solution was irradiated with 340 nm light, the maxima at *λ* = 331 and 347 nm decreased and slightly shifted bathochromically.^19^ Furthermore, an absorption shoulder band emerged around *λ* = 365 nm. These UV-vis spectral changes were indicative of the transformation of the stiff-stilbene (*E*)-isomer to the (*Z*)-isomer.^9,10^ The sample was irradiated until no further spectral changes were observed, *i.e.* the photostationary state (PSS) was reached. The reverse spectral changes were observed upon irradiation with 365 nm light to regenerate the (*E*)-bisCP isomer. In both cases, a clear isosbestic point was maintained at *λ* = 355 nm, indicating unimolecular conversion (Fig. S26 and S27 in the ESI†).

The photoisomerization process was additionally investigated by ^1^H NMR spectroscopy. Upon exposure of a solution of (*E*)-bisCP in 0.5 vol% H_2_O/DMSO-*d*_6_ (*c* = 0.5 mM) to 340 nm light, an additional set of proton signals became present, which could be assigned to (*Z*)-bisCP (Fig. 1B and S28 in the ESI†). For example, alongside the aromatic proton signals (doublet and singlet at *δ* = 8.47 and 7.94 ppm, red) characteristic of the stiff-stilbene (*E*)-isomer, two new aromatic proton signals (at *δ* = 8.40 and 8.23 ppm, blue) appeared. Integration of the ^1^H NMR signals at the point where PSS had been reached gave an isomer ratio of 50 : 50. When the same sample was subsequently irradiated with 365 nm light, the proton signals of (*Z*)-bisCP disappeared and the original ^1^H NMR spectrum of (*E*)-bisCP was recovered, which is in line with what was observed by UV-vis spectroscopy.

### Sulfate complexation studies

**Fig. 2:**
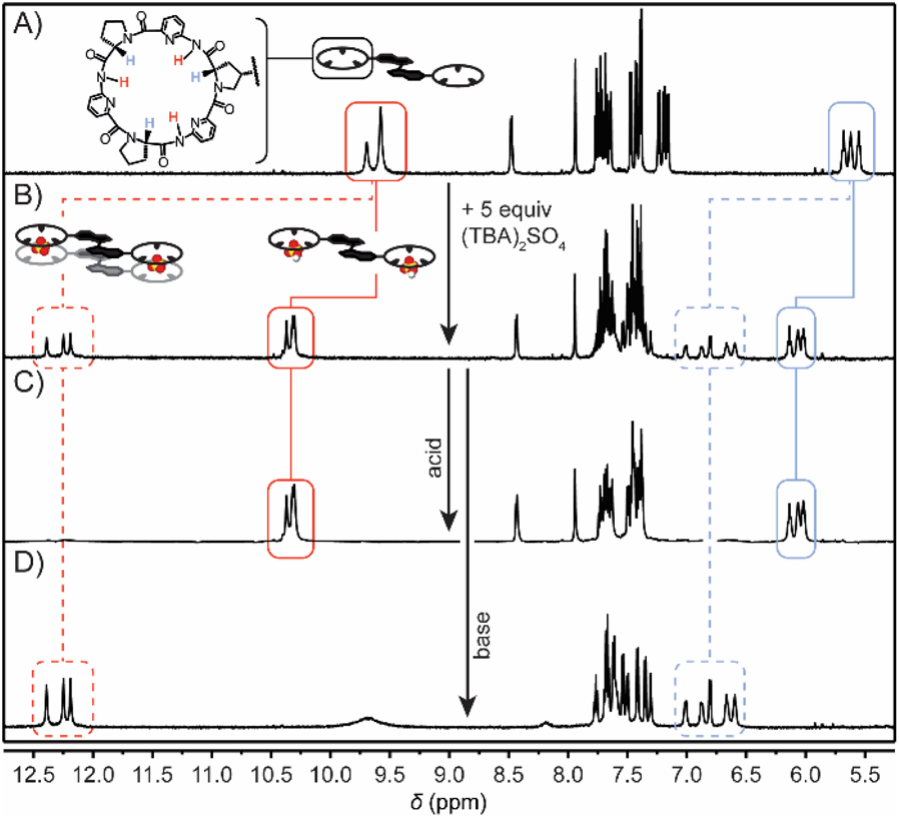
^1^H NMR spectra of (A) (*E*)-bisCP in 0.5 vol% H_2_O/DMSO-*d*_6_, (B) after the addition of 5 equiv. of TBA_2_SO_4_, (C) after the addition of acid (H_2_SO_4_) to exclusively form (*E*)-bisCP·(HSO_4_^−^)_2_, and (D) after the addition of base (DIPEA) to exclusively form [(*E*)-bisCP·SO_4_^2−^]_2_.

The interaction between the bis(cyclopeptide) isomers and SO_4_^2−^ was initially studied by ^1^H NMR titrations in 0.5 vol% H_2_O/DMSO-*d*_6_.^18^ In the ^1^H NMR spectrum of (*E*)-bisCP, the most conclusive signals for monitoring sulfate complexation are the broad singlets of the NH protons (*δ* = 9.88–9.64 ppm, Fig. 2A), due to their active involvement in anion–hydrogen bonding. In addition, the H(α) protons of the l-proline unit, which give three broad triplets (*δ* = 5.75–5.52 ppm) in the uncomplexed form, are oriented close to the anion binding site and will be substantially deshielded upon interaction with sulfate.^7*a*,8^

Following the stepwise addition of (TBA)_2_SO_4_ (TBA = tetrabutylammonium) to (*E*)-bisCP until 5 equiv., these two sets of signals gradually shifted downfield (to *δ* = 10.45–10.30 and to 6.21–6.00 ppm, respectively, see Fig. 2B and S8 in the ESI†), which suggests the formation of a complex with fast binding kinetics. Unexpectedly, at around 0.5 equiv. of SO_4_^2−^, an additional set of signals became visible, including three sharp singlets for the NH protons, which were shifted even further downfield (*δ* = 12.19, 12.25 and 12.40 ppm), whereas the signals for the cyclopeptide H(α) protons were also further downfield (*δ* = 7.04–6.54 ppm). The chemical shift of these signals was almost unaffected by the (TBA)_2_SO_4_ concentration, indicating the formation of a species in slow exchange on the NMR timescale. Another (presumably more stable) complex thus additionally formed in the mixture. We hypothesized that the former shifting of signals could be due to complexation with HSO_4_^−^ anions, likely formed by proton transfer from water molecules to (TBA)_2_SO_4_ due to the basicity of SO_4_^2−^ in DMSO.^14^ We ascribed the latter, more downfield shifted signal set to an SO_4_^2−^ complex, since the double negative charge should cause more pronounced proton deshielding and more efficient interactions with the bis(cyclopeptide). These assignments were supported by the fact that the use of TBA(HSO_4_) instead of (TBA)_2_SO_4_ caused the relative amount of the presumed SO_4_^2−^ complex at the end of the titration to decrease from 35% to 28% (Fig. S9 in the ESI†). Furthermore, this relative amount slightly dropped beyond the addition of 1 equiv., which could be an indication that the SO_4_^2−^ and HSO_4_^−^ complexes of (*E*)-bisCP have distinct stoichiometry (Fig. S10 in the ESI†). Finally, modified Job plot analysis using the gradually shifted signals hinted at a 1 : 2 (*E*)-bisCP/HSO_4_^−^ complex stoichiometry (see Fig. S10 in the ESI†).

An analogous observation was made when the non-substituted parent cyclopeptide (CP) as a model compound was titrated with TBA(HSO_4_) to also afford a mixture of fast- and slow-exchanging complexes of which the relative ratio changed throughout the titration. In this case, modified Job plot analysis using the gradually shifted signals was in closer agreement with the monotopic cyclopeptide's expected 1 : 1 complexation (Fig. S20 and S21 in the ESI†). Notably, when either (*E*)-bisCP or the parent CP was titrated with an exclusively mono-anionic guest, *i.e.* bromide, only gradual downfield shifting of the ^1^H NMR signals was observed (Fig. S16 and S22,† respectively). Moreover, these chemical shift changes were very similar to those allocated to HSO_4_^−^ complex formation. Fitting of this titration data to 1 : 2 and 1 : 1 models for (*E*)-bisCP and parent CP, respectively, afforded a log *K*_a_ of ∼3 for each bromide binding event (Fig. S17 and S23†).

Further evidence for the existence of protonation-dependent complexes was obtained by performing acid–base titrations. To this end, an aliquot (0.5 mL) of a solution of (*E*)-bisCP in the presence of 5 equiv. of (TBA)_2_SO_4_ was taken and titrated with minute quantities of H_2_SO_4_ (50 mM) (Fig. 2C and S11 in the ESI†). This titration eventually led to exclusive formation of the presumed HSO_4_^−^ complex at the expense of the SO_4_^2−^ complex. In a separate experiment, small amounts of *N*,*N*-diisopropyl-ethylamine (DIPEA) were added to the (*E*)-bisCP/(TBA)_2_SO_4_ mixture, which caused the disappearance of all peaks related to the HSO_4_^−^ complex in favour of the peaks belonging to the SO_4_^2−^ complex (Fig. 2D and S12 in the ESI†). In addition, when the entire ^1^H NMR titration of (*E*)-bisCP with (TBA)_2_SO_4_ was performed in the presence of 0.05 vol% of DIPEA, only the signals of the slow-exchanging species, assigned to the SO_4_^2−^ complex, were observed (Fig. S13 in the ESI†). Importantly, this titration saturated at a 1 : 1 stoichiometry of (*E*)-bisCP and the SO_4_^2−^ dianion.

^1^H NMR titrations in which TBAHSO_4_ or TBA_2_SO_4_ was added to a solution of (*Z*)-bisCP were also performed (as previously, the titration with TBA_2_SO_4_ contained additional 0.05 vol% DIPEA to ensure complete deprotonation of SO_4_^2−^). In both titrations, the broad singlet signals of the NH protons shifted downfield (from *δ* = 9.75–9.50 ppm to *δ* = 12.38–12.08) and appeared as three sharp singlets (Fig. S14 and S15 in the ESI†). Analogously, the broad signals of the H(α) proton of the l-proline unit shifted downfield (from *δ* = 5.75–5.50 ppm to *δ* = 6.75–6.55 ppm). These chemical shifts confirm the complexation of both anions. Furthermore, the titrations saturated at 1 equiv. of anion and the exchange was slow on the NMR timescale, suggesting efficient 1 : 1 binding due to the good preorganization of the receptor.

To determine what type of complex formed, ESI mass spectra of solutions of (*E*)-bisCP and (*Z*)-bisCP in 5 vol% H_2_O/CH_3_OH containing 1 equiv. of Na_2_SO_4_ were recorded.^19^ The spectrum of (*Z*)-bisCP in the negative mode featured mainly one signal whose *m*/*z* ratio and isotopic pattern were consistent with the expected doubly charged 1 : 1 complex (*Z*)-bisCP·SO_4_^2−^ (*m*/*z* calcd 856.79, exp. 856.79, see Fig. S24 in the ESI†). In addition, a weak signal was visible at *m*/*z* = 1735.41, which could be attributed to the singly charged sodium adduct of the 1 : 1 complex (*m*/*z* calcd 1735.56, exp. 1735.41). The ESI mass spectrum of (*E*)-bisCP also contained a major signal at *m*/*z* = 856.79. However, in contrast to the spectrum of (*Z*)-bisCP, the individual lines were separated by only 0.25 *m*/*z* units, indicating that this signal corresponded to a fourfold negatively charged complex with the composition [(*E*)-bisCP·SO_4_^2−^]_2_ (Fig. S25 in the ESI†). A smaller signal in the spectrum appeared at the *m*/*z* ratio of the corresponding triply charged sodium adduct.

These mass spectra confirm that the convergent arrangement of the two cyclopeptide rings in (*Z*)-bisCP allowed them to engage simultaneously in anion binding, as in other bis(cyclopeptides) containing flexible linkers.^8,20^ Unexpectedly, however, the divergent arrangement in (*E*)-bisCP induced by the rigid stiff-stilbene linker did not prevent the cyclopeptides from forming sandwich complexes, but gave rise to a previously unobserved binding mode in which two (*E*)-bisCP units bridged two anions.

**Fig. 3:**
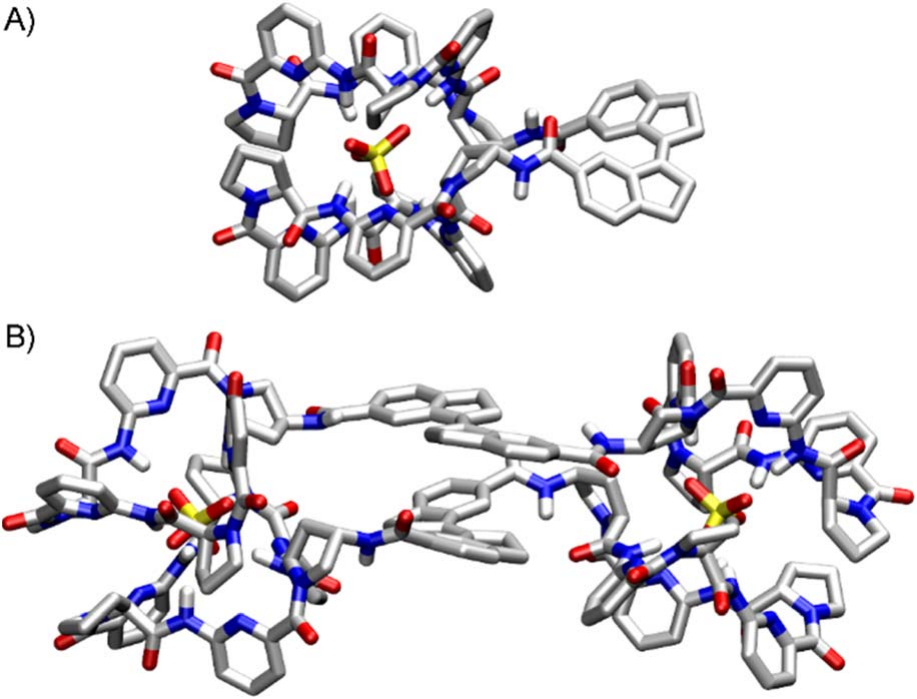
Calculated structures of the 1 : 1 [(*Z*)-bisCP·SO_4_^2−^] complex (A) and the 2 : 2 [(*E*)-bisCP]_2_·(SO_4_^2−^)_2_ complex (B). All but the NH protons are omitted for reasons of clarity. The calculations were performed by using the MMFF force field followed by a DFT optimisation (B3LYP/6-31G*) of the obtained structures by using Spartan 24 (Wavefunction, Inc.).

DFT calculations were performed to visualize the arrangements of the bis(cyclopeptides) in these complexes. These calculations were based on the crystal structures of the 1 : 2 sandwich complex between iodide and CP as well as the 1 : 1 sulfate complex of a bis(cyclopeptide) with a flexible linker.^7*b*,21^ As can be seen in Fig. 3, the stiff-stilbene linker in (*Z*)-bisCP induces an orientation of the two cyclopeptide rings that allows them to simultaneously bind the sulfate anion in an apparently strain-free manner, resulting in a complex structure that is closely related to that observed for another bis(cyclopeptide).^21^ In (*E*)-bisCP, on the other hand, the formation of the characteristic sandwich-type complexes requires two stiff-stilbene linked bis(cyclopeptide) molecules to come together. In the corresponding 2 : 2 complex, the two stiff-stilbene units are aligned almost perfectly. Aromatic stacking interactions, reinforced by the desolvation of the hydrophobic surfaces, are therefore likely to contribute to the stability of the 2 : 2 complex.

Since the complexation kinetics of both these SO_4_^2−^ complexes were slow on the NMR timescale, we resorted to isothermal titration calorimetry (ITC) to quantify their stability. Therefore, a solution of TBAHSO_4_ in 0.5 vol% H_2_O/0.05 vol% DIPEA/DMSO was titrated to a solution of the bis(cyclopeptides) in the same solvent mixture. As described for the ^1^H NMR experiments, the presence of the base ensures that only the SO_4_^2−^ complexes can form in solution. In the case of (*E*)-bisCP, exothermic heat pulses were observed, and a steep binding isotherm suggested strong and highly cooperative binding. Fitting this isotherm to the mathematical model of a 1 : 1 complex gave an inflection point *n* of 0.8, close to the inflection point of 1 expected for a 2 : 2 complex (Fig. S39 in the ESI†). A reference measurement using the monotopic unsubstituted cyclopeptide as host resulted in a similar isotherm, but this time with an inflection point at 0.5, indicative of the formation of a sandwich-type 2 : 1 host–guest complex. Due to the high steepness of the binding isotherms and, in the case of (*E*)-bisCP, the complexity of the 2 : 2 binding model, the stability constants could not be derived. Moreover, competitive titrations were not performed because isotherms afforded by titrations of other anions (halides and nitrate) could not be fitted. Nonetheless, the isotherms for the titration with SO_4_^2−^ still provide evidence that the interaction with (*E*)-bisCP is very efficient (log *K*_a_ > 7).

The titration with (*Z*)-bisCP under similar conditions also gave a steep binding isotherm with an inflection point of 0.8. This time, however, we were able to first determine the stability of the Br^−^ complex of the bis(cyclopeptide) by using TBABr as guest salt (Fig. S40 in the ESI†). The log *K*_a_ of 4.1 hence obtained by ITC was in excellent agreement with that determined independently by an NMR titration (Fig. S18 and S19 in the ESI†). This stability constant allowed us to perform a competitive ITC titration by adding TBA_2_SO_4_ to the Br^−^ complex of (*Z*)-bisCP (Fig. S40 in the ESI†). The resulting isotherm was fitted to the one-site binding model, yielding a log *K*_a_ of 9.0 for the SO_4_^2−^ complex of (*Z*)-bisCP, which was slightly larger than the log *K*_a_ observed for a doubly-linked bis(cyclopeptide) in 67 vol% acetonitrile/water.^22^

We also attempted to quantify the stability of the HSO_4_^−^ complexes by titrating a solution of TBAHSO_4_ in 0.5 vol% H_2_O/DMSO into solutions of (*E*)- and (*Z*)-bisCP in the same solvent mixture. For both isomers, weak heat pulses were observed, which in the case of (*E*)-bisCP resulted in a flat binding isotherm that could not be reliably fitted (Fig. S41 in the ESI†). In the case of (*Z*)-bisCP, the binding isotherm was discontinuous. The difficulties encountered in these measurements might be due to the additional acid–base equilibrium between SO_4_^2−^ and HSO_4_^−^. Nonetheless, the significantly different shapes of the observed binding isotherms provided strong evidence that the HSO_4_^−^ complexes of both bis(cyclopeptides) were significantly less stable than the SO_4_^2−^ complexes. This lower stability is reasonable, since HSO_4_^−^ exhibits a lower charge and repulsive interactions can occur between NH groups and the proton on the anion.

### Light-controlled complexation

**Fig. 4:**
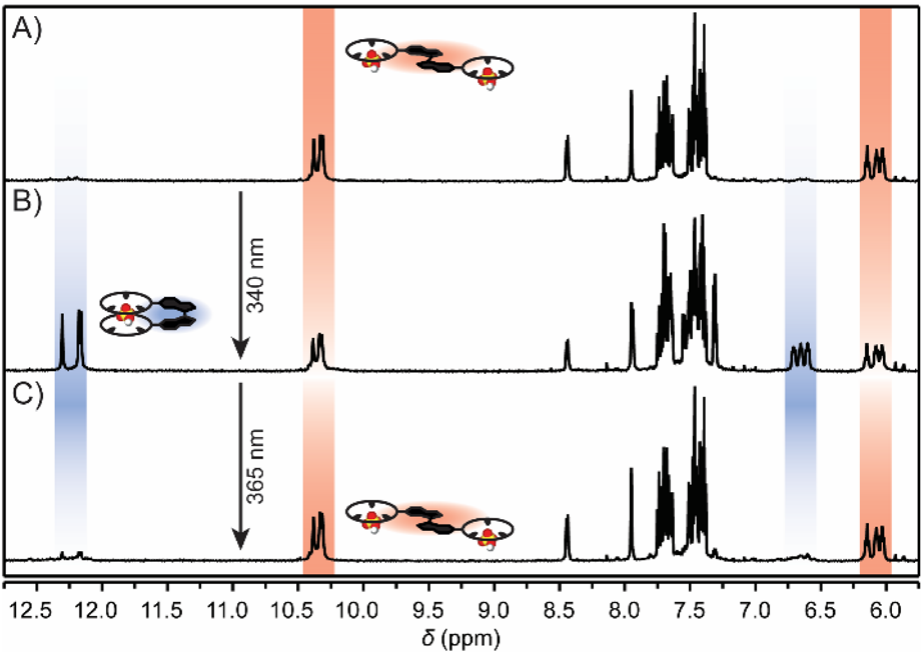
^1^H NMR spectral changes of (A) (*E*)-bisCP in 0.5 vol% H_2_O/DMSO-*d*_6_ (0.5 mM) in the presence of an excess of TBAHSO_4_ (2.5 mM) after irradiation with (B) 340 nm light and (C) consecutive irradiation with 365 nm light at 20 °C.

As shown above, 1 : 2 and 2 : 2 complexation modes of (*E*)-bisCP can be accessed by addition of acid and base, respectively, while (*Z*)-bisCP is always a 1 : 1 complexed species, regardless of the sulfate protonation state. We additionally sought to isomerize the bis(cyclopeptide) in the presence of HSO_4_^−^ and SO_4_^2−^ such that the 1 : 2 and 2 : 2 complexes would interconvert with the 1 : 1 complex *in situ* (see Scheme 1). Hence, the effect of irradiation on the NH and proline H(α) proton resonances, which are most sensitive to the configuration of the bis(cyclopeptide) and their interaction with anions, was studied by ^1^H NMR spectroscopy. As described above, the signals of these protons appear between *δ* = 10.5 and 10.3 ppm (NH) and between *δ* = 6.3 and 6.0 ppm [H(α)] in the spectrum of a solution of (*E*)-bisCP in 0.5 vol% H_2_O/DMSO-*d*_6_ (0.5 mM) containing 5 equiv. of TBAHSO_4_ (Fig. 4A). Under these conditions, the 1 : 2 [(*E*)-bisCP·(HSO_4_^−^)_2_] complex was predominantly present. When this solution was irradiated with 340 nm light, a reduction in peak intensity of these characteristic signals was observed as the bisCP switched from the (*E*)- to the (*Z*)-configuration. At the same time, three new sharp singlets appeared at *δ* = 12.31, 12.18, and 12.16 ppm, which correspond to the NH proton signals of (*Z*)-bisCP·HSO_4_^−^ (Fig. 4B and S29 in the ESI†). Integration of the different ^1^H NMR signal sets gave a PSS_340_ (*E*/*Z*) ratio of 46 : 54, which is thus minimally affected by the presence of the anion [*i.e.* PSS_340_ (*E*/*Z*) = 50 : 50 in the absence of HSO_4_^−^, see above]. Next, the solution was irradiated at 365 nm to reverse the configurational change (Fig. 4C). Upon this sequential irradiation, the NH signals of the 1 : 1 [(*Z*)-bisCP·HSO_4_^−^] complex disappeared almost fully, while the NH proton signals of the 1 : 2 [(*E*)-bisCP·(HSO_4_^−^)_2_] complex regained their original intensity. These results show that 1 : 2 and 1 : 1 HSO_4_^−^ complexes of (*E*)-bisCP and (*Z*)-bisCP, respectively, can be successfully interconverted by light.

Next, we investigated the effect of irradiation on the 2 : 2 [(*E*)-bisCP_2_·(SO_4_^2−^)_2_] complex. To this end, a solution of (*E*)-bisCP in a mixture of 0.5% vol% H_2_O/0.05% vol% DIPEA/DMSO-*d*_6_ (0.5 mM) in the presence of 5 equiv. of TBA_2_SO_4_ was prepared. When the sample was irradiated with 340 nm light at 20 °C, little *E*-to-*Z* isomerization was observed. Instead, new signals appeared in the ^1^H NMR spectrum (Fig. S30 in the ESI†), which became more pronounced when the irradiation wavelength was changed to 365 nm. The same signals appeared when a sample of the 1 : 1 [(*Z*)-bisCP·(SO_4_^2−^)] complex was irradiated at 365 nm. Here, irradiation was continued until the ^1^H NMR spectrum indicated complete conversion (Fig. S35 in the ESI†). A mass spectrum (positive mode) was then recorded in which a peak at *m*/*z* = 825.31 indicated the presence of monotopic peptides containing an appended indanone residue (Fig. S36 and S37 in the ESI†). This residue is known to be formed during photo-oxidation of stiff-stilbene,^23^ and the bis(cyclopeptide) was therefore apparently cleaved at the double bond upon irradiation.

**Fig. 5:**
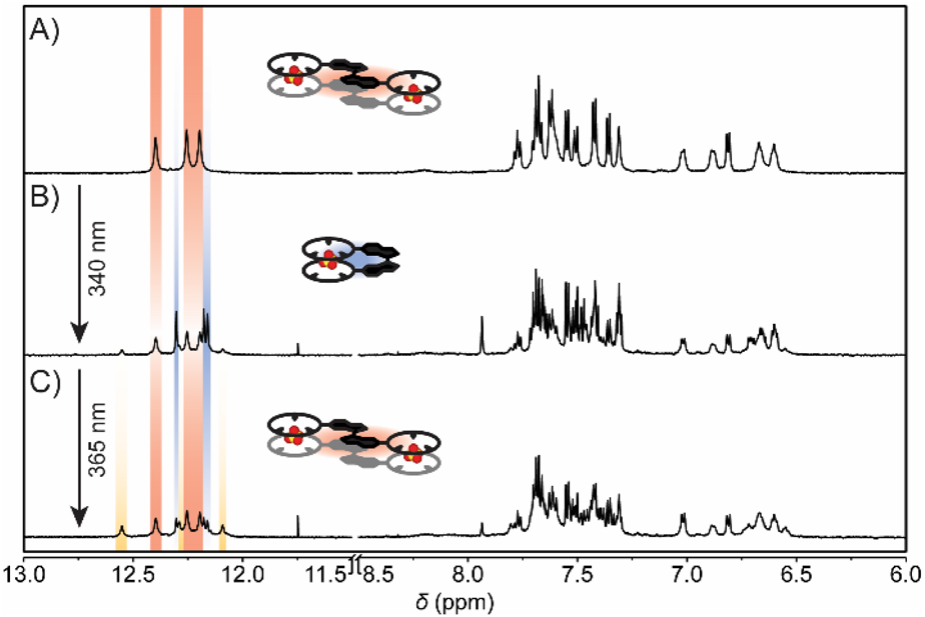
^1^H NMR spectral changes of (A) (*E*)-bisCP in 0.5 vol% H_2_O/0.05 vol% DIPEA/DMSO-*d*_6_ (0.5 mM) in the presence of TBASO_4_ (2.5 mM) after irradiation with (B) 340 nm light and (C) consecutive irradiation with 365 nm light at 160 °C.

We hypothesized that isomerization of the SO_4_^2−^ complexes is impeded because of their high stabilities. Hence, to weaken the binding interactions, irradiation was performed additionally at 160 °C (Fig. 5 and S32 in the ESI†). When the solution of the 2 : 2 [(*E*)-bisCP_2_·(SO_4_^2−^)_2_] complex was irradiated with 340 nm light, the respective NH proton signals shifted from *δ* = 12.40, 12.26, and 12.20 ppm to *δ* = 12.30, 12.18, and 12.16 ppm as a sign of conversion to the 1 : 1 [(*Z*)-bisCP·SO_4_^2−^] complex (Fig. 5A and B). Eventually, a PSS was reached with a ratio of 38 : 62 (*E*/*Z*), which is significantly higher than the 50 : 50 ratio obtained for the uncomplexed bis(cyclopeptide). Consecutive irradiation of the PSS_340_ mixture with 365 nm light resulted in recovery of the 2 : 2 [(*E*)-bisCP_2_·(SO_4_^2−^)_2_] complex, although partial decomposition was observed (∼4%, signals at *δ* = 12.55, 12.29, and 12.09 ppm, Fig. 5C). It should be noted that in the presence of a weaker binding anion such as bromide, seamless photoswitching was observed (Fig. S31 in the ESI†). The observed decomposition at lower temperature to the indanone product (Fig. S30, S33 and S34†) thus indeed seems to be due to the tight binding of SO_4_^2−^.

Overall, the above findings confirm that switching between the 2 : 2 sandwich complex and the 1 : 1 complex is feasible, even though traces of the indanone product were detected when (*Z*)-bisCP·SO_4_^2−^ was irradiated back to [(*E*)-bisCP·SO_4_^2−^]_2_ at 160 °C. Advantageously, photoisomerization not only becomes viable by raising the temperature, but also by protonation of the SO_4_^2−^ dianion since conversion in the presence of the HSO_4_^−^ anion was shown to be clean. Accordingly, interconversion between the [(*E*)-bisCP·SO_4_^2−^]_2_ and (*Z*)-bisCP·SO_4_^2−^ complex is possible by first protonating the anion, followed by irradiation at 20 °C and subsequent addition of base to deprotonate the anion. The different switching pathways are summarized in Scheme 1.

## Conclusion

A bis(cyclopeptide) containing a rigid stiff-stilbene linker can be isomerized by light between its (*E*)- and (*Z*)-configuration. In the (*Z*)-form, this compound forms 1 : 1 sandwich-type complexes with hydrogen sulfate (HSO_4_^−^) as well as the sulfate dianion (SO_4_^2−^) *via* simultaneous interaction with both cyclopeptide anion-binding moieties. In the (*E*)-form, in which the cyclo-peptides are further away from each other, 1 : 2 receptor/HSO_4_^−^ complexation was observed, while with SO_4_^2−^ a 2 : 2 complex was found. Photoisomerization allows interconversion between the respective 1 : 1/1 : 2 and 1 : 1/2 : 2 complexes for HSO_4_^−^ and SO_4_^2−^. Protonation of the SO_4_^2−^ dianion, which weakens the complex, provides an attractive orthogonal stimulus to switch between the 1 : 2 and 2 : 2 complexed states. Our results demonstrate how the structure and composition of anion–ligand complexes can be controlled by two types of stimuli. We predict that in the future different (and more sophisticated) types of nanostructures can be accessed by introducing other anions. The presented dual-stimulus control would then allow, for example, the encapsulation and release of cargo molecules or activation of catalysis on demand. Work in this direction is currently ongoing in our laboratories.

## Data availability

The data supporting this article have been included as part of the ESI.†

## Author contributions

S. M., B. W., S. K., and S. J. W. conceived the project. S. M. and B. W. synthesized the materials. S. M. carried out the UV-vis and ^1^H NMR photoisomerization studies. S. M. and J. E. B. conducted the ^1^H NMR titrations. B. W. prepared the ESI mass spectra in the aqueous solvent mixture and performed the ITC experiments. S. K. performed the DFT calculations. S. M., S. K. and S. J. W. wrote the manuscript. The project was guided by S. K. and S. J. W.

## Conflicts of interest

There are no conflicts to declare.

## Supplementary Material

SC-016-D5SC00766F-s001

SC-016-D5SC00766F-s002

SC-016-D5SC00766F-s003
