# Supplementary material for: Light and protonation-controlled complex formation between sulfate ions and a stiff-stilbene based bis(cyclopeptide)
Source: Chem Sci. 2025 Mar 24;16(18):7822–8. doi: 10.1039/d5sc00766f (PMC11959293; doi:10.1039/d5sc00766f)
Supplement: SC-016-D5SC00766F-s001 [file SC-016-D5SC00766F-s001.pdf]

Supplementary Information (SI) for Chemical Science.
This journal is © The Royal Society of Chemistry 2025

ELECTRONIC SUPPLEMENTARY INFORMATION (ESI)

Light and protonation-controlled complex formation between sulfate ions and a stiff-stilbene based bis(cyclopeptide)

Stefan Mommer,[a,‡] Benedict Wyrwol,[b] Jasper E. Bos,[a]
Stefan Kubik*[b] and Sander J. Wezenberg*[a]

[a] Leiden Institute of Chemistry, Leiden University,
Einsteinweg 55, 2333 CC Leiden, The Netherlands

[b] Fachbereich Chemie – Organische Chemie, RPTU Kaiserslautern-Landau,
Erwin-Schrödinger-Str. 54, 67663 Kaiserslautern, Germany

‡ Present address: Macromolecular Engineering Laboratory, ETH Zurich,
Sonneggstrasse 3, 8092, Zurich, Switzerland.

Email: stefan.kubik@rptu.de; s.j.wezenberg@lic.leidenuniv.nl

Table of Contents

1. Experimental section S2
2. ^{1}H NMR spectroscopic titrations S10
3. Mass spectrometric binding studies S20
4. UV-vis photoisomerisation studies S22
5. ^{1}H NMR photoisomerisation studies S24
6. ITC titrations S33
7. DFT Calculations S38
8. References S39

1. Experimental section

1.1. Materials

The (*E*)-isomer of the stiff-stilbene bis(carboxylic acid) was prepared using a protocol by Akbulatov *et al.*,[1] while the corresponding (*Z*)-isomer was obtained according to a procedure reported by Villarón *et al.*[2] *N*,*N*-Diisopropylethylamine (DIPEA, 99.5%, Acros Organics), 2-(1H-benzotriazole-1-yl)-1,1,3,3-tetramethylaminium tetrafluoroborate (TBTU, 98%, Sigma-Aldrich), tetrabutylammonium bromide (TBABr, >98%, Sigma-Aldrich), tetrabutylammonium hydrogen sulfate $TBAHSO_4$, 99%, Acros Organics), bis(tetrabutylammonium) sulfate solution (TBA_2SO_4, ~50% in H_2O, Sigma-Aldrich) were used without further purification. Unless otherwise noted, solvents were purchased from commercial sources and were used without further purification.

1.2. Instrumentation

^{1}H and ^{13}C NMR spectra were recorded on Bruker Avance 400, 500 or 600 NMR spectrometer (400/101 MHz, 500 MHz/126 MHz, 600 MHz/151 MHz, respectively) and chemical shifts are reported as follows: δ (ppm) (multiplicity, coupling constant J (Hz), number of protons, assignment). The residual solvent signals (DMSO, δ_H = 2.50 ppm, δ_C = 39.5 ppm) were used as reference. Chemical shifts are reported in ppm to the nearest 0.01 ppm for ^{1}H and the nearest 0.1 ppm for ^{13}C. Irradiation of samples was carried out using Thorlab model M340F3 (0.85 mW), M365F1 (4.1 mW) and M385F1 (9.0 mW), positioned at a distance of 5 cm to the sample, unless noted otherwise.

UV-vis spectra were recorded on an Agilent Cary 8454 spectrometer using 1 cm or 1 mm quartz cuvettes. Irradiation of samples was carried out using Thorlab model M340F3 (0.85 mW), M365F1 (4.1 mW) and M385F1 (9.0 mW), positioned at a distance of 1 cm to the sample, unless noted otherwise.

High-resolution mass spectrometry (HRMS) was performed on a Thermo Scientific Q Exactive HF spectrometer with electron spray ionisation (ESI). For the mass spectrometric binding studies, a Bruker Daltronics Esquire 3000 instrument was used. The spectra were exported as ASCII files and further processed with ProFit 7, Quantum Soft, Switzerland.

ITC measurements were performed at 25 °C by using a Microcal VP-ITC (see section 6 for details).

1.3. Syntheses

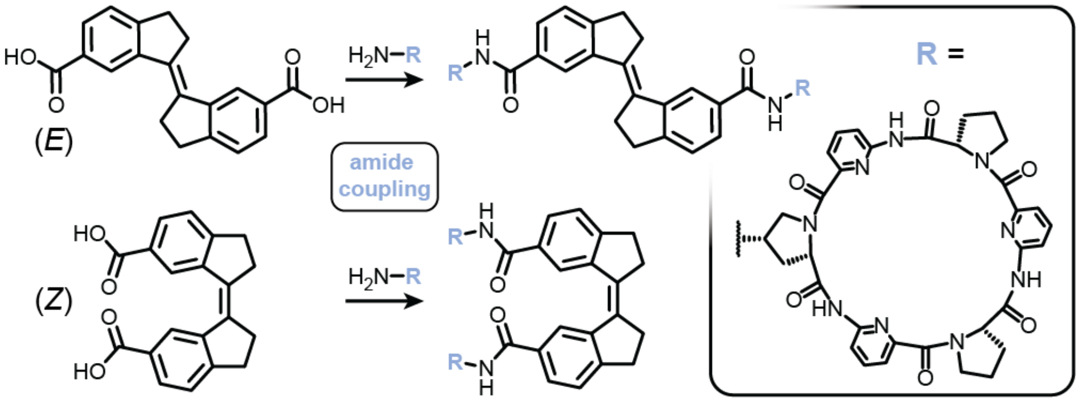

Figure S1. Synthesis of (*E*)- and (*Z*)-bis**CP** starting from the stiff-stilbene bis(carboxylic acid)s. Coupling conditions: (*E*/*Z*)-stiff-stilbene bis(carboxylic acid) (1 equiv.), cyclopeptide mono-amine (2 equiv.), TBTU (2 equiv.), *N*,*N*-diisopropylethylamine (10 equiv.), DMF, 25 °C, 16 h.

Bis(cyclopeptide) with (*Z*)-configured linker [(*Z*)-bis**CP**]

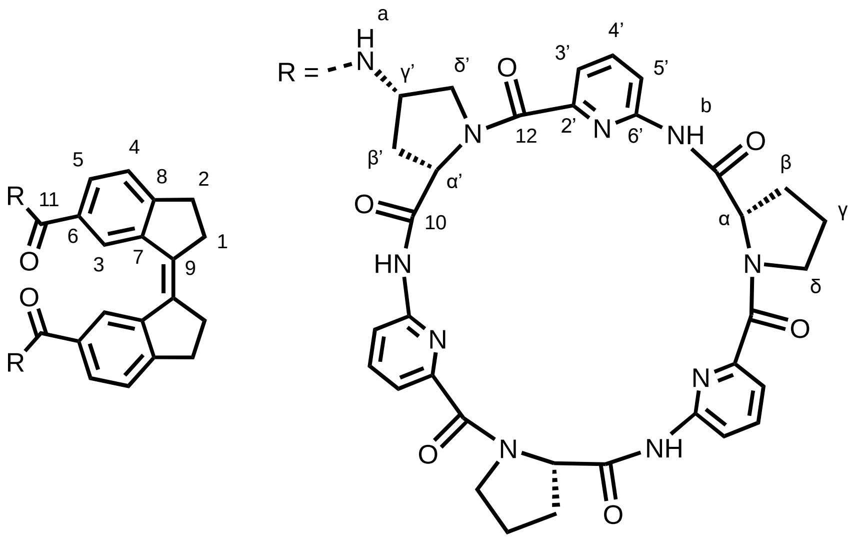

The synthesis started from the Cbz-protected version of a cyclic hexapeptide with one 4-aminoproline residue whose synthesis has been described elsewhere.[3] The corresponding unprotected monoamine was obtained by dissolving this cyclopeptide (134 mg, 167 µmol) in dichloromethane/methanol 1:1 (*v*/*v*) (50 mL), followed by the addition of 10% Pd/C (18 mg), $Pd(OH)_2$/C (18 mg) and 1 M aqueous HCl (184 µL, 184 µmol). The reaction mixture was stirred for 4 d at 25 °C under an atmosphere of hydrogen, the catalysts were removed by filtration through celite, and the filtrate was evaporated to dryness. The thus obtained colorless solid was used in the next step without further purification. Yield: 105 mg (149 µmol, 89%).

The product (105 mg, 149 μmol, 2 equiv), the (*Z*)-isomer of the stiff stilbene bis(carboxylic acid) (24 mg, 75 μmol, 1 equiv), and TBTU (48 mg, 149 μmol, 2 equiv) were dissolved in DMF (4 mL) under an atmosphere of nitrogen in the dark. DIPEA (130 μL, 750 μmol, 10 equiv) was added, and the mixture was stirred at room temperature for 16 h. The solution was evaporated to a volume of 1 mL, water was added, and the precipitate was filtered off. The solid obtained was washed with water, dried, and dissolved in methanol/DMF, 10:1 (*v/v*). Pure product was isolated by preparative HPLC (Dionex UltiMate 3000; column, Thermo Fisher, BetaBasic-18, 250 × 21.2 mm, 5 μm particle size; flow, 10 mL/min, T = 25 °C) using water/acetonitrile mixtures as eluents and the following gradient: 0 min, 10 vol% acetonitrile; 0-30 min, linear increase to 90 vol% acetonitrile; 30-32 min, 90 vol% acetonitrile; 32–33 min, linear decrease to 10 vol% acetonitrile; 33-35 min, 10 vol% acetonitrile. The product eluted between minutes 21.10 and 22.65. The fractions containing the product were pooled, the organic solvent removed *in vacuo*, and the remaining water layer freeze-dried to obtain the product as a cream-colored solid. Yield: 39 mg (24 μmol, 32%); m.p. > 250 °C (dec.); ^{1}H NMR (400 MHz, 25 °C, DMSO-d_6): δ 9.60 (s, 2H, NH^{b}), 9.55 (s, 2H, NH^{b}), 9.56 (s, 2H, NH^{b}), 8.40 (s, 2H, H3), 8.24 (d, J = 6.1 Hz, 2H, NH^{a}), 7.70 (t, J = 7.7 Hz, 4H, H4'), 7.62 – 7.57 (m, 4H, H4'/H5), 7.44 – 7.40 (m, 4H, H5'), 7.35 – 7.33 (m, 4H, H5'/H4), 7.25 (d, J = 8.2 Hz, 2H, H3'), 7.14 (d, J = 8.2 Hz, 2H, H3'), 7.09 (d, J = 8.2 Hz, 2H, H3'), 5.64 (t, J = 7.3 Hz, 2H, Hα'), 5.59 – 5.53 (m, 4H, Hα), 4.67 – 4.58 (m, 2H, Hγ'), 3.94 – 3.90 (m, 2H, Hδ'), 3.72 – 3.62 (m, 6H, Hδ/Hδ'), 3.61 – 3.51 (m, 4H, Hδ), 3.00 – 2.94 (m, 4H, H1), 2.91 – 2.82 (m, 2H, Hβ'), 2.82 – 2.75 (m, 4H, H2), 2.59 – 2.50 (m, 4H, Hβ), 2.31 – 2.21 (m, 2H, Hβ'), 2.08 – 1.97 (m, 4H, Hβ), 1.89 – 1.77 (m, 8H, Hγ) ppm; ^{13}C NMR (151 MHz, 25 °C, DMSO-d_6): δ 171.0 (C10), 170.7 (C10), 166.5 (C11), 165.9 (C12), 152.0 (C6'), 151.8 (C6'), 151.6 (C6'), 151.3 (C8), 148.5 (C2'), 139.6 (C7), 139.0 (C4'), 138.8 (C4'), 134.7 (C9), 131.9 (C6), 126.5 (C5), 125.0 (C3), 121.8 (C4), 119.7 (C5'), 119.5 (C5'), 115.9 (C3'), 115.8 (C3'), 115.5 (C3'), 61.5 (Cα), 60.5 (Cα'), 51.8 (Cδ'), 48.2 (Cδ), 47.0 (Cγ'), 37.2 (Cβ'), 34.1 (C1), 30.0 (C2), 22.4 (Cγ) ppm; MS (MALDI-TOF, positive mode) *m/z* (%): 1602.6 $[M–O+H]^+$ (22), 1617.7 $[M+H^+]$ (36), 1639.7 $[M+Na^+]$ (100), 1655.7 $[M+K^+]$ (29); CHN calculated for $C_{86}H_{80}N_{20}O_{14}\cdot 10\ H_2O$ (M.W.: 1797.84): C, 57.45%; H, 5.58%; N, 15.58%; found: C, 57.69%; H, 5.27%; N, 15.31%.

Bis(cyclopeptide) with (*E*)-configured linker [(*E*)-bis**CP**]

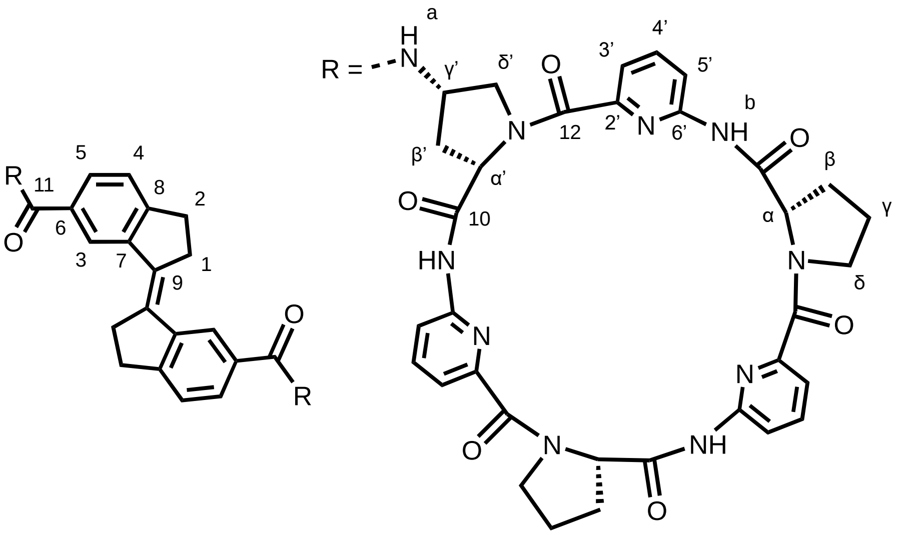

The Cbz-protected cyclopeptide (119 mg, 149 µmol) also used for the synthesis of (*Z*)-bis**CP** was dissolved dichloromethane/methanol 1:1 (*v/v*) (50 mL), followed by the addition of 10% Pd/C (15 mg), $Pd(OH)_2$/C (15 mg) and 1 M aqueous HCl (164 µL, 164 µmol). The reaction mixture was stirred for 4 d at 25 °C under an atmosphere of hydrogen, the catalysts were removed by filtration through celite, and the filtrate was evaporated to dryness. The thus obtained colorless solid was used in the next step without further purification. Yield: 85 mg (121 µmol, 81%).

The product (121 mg, 121 µmol, 2 equiv), (*E*)-isomer of the stiff stilbene bis(carboxylic acid) (19 mg, 60 µmol, 1 equiv), and TBTU (39 mg, 121 µmol, 2 equiv) were dissolved in DMF (4 mL) under an atmosphere of nitrogen in the dark. DIPEA (105 µL, 600 µmol, 10 equiv) was added, and the mixture was stirred at room temperature for 16 h. The solution was evaporated to a volume of 1 mL, water was added, and the precipitate was filtered off. The solid obtained was washed with water, dried, and dissolved in methanol/DMF, 10:1 (*v/v*). Pure product was isolated by preparative HPLC (Dionex UltiMate 3000; column, Thermo Fisher, BetaBasic-18, 250 × 21.2 mm, 5 µm particle size; flow, 10 mL/min, T = 25 °C) using water/acetonitrile mixtures as eluents and the following gradient: 0 min, 10 vol% acetonitrile; 0-30 min, linear increase to 90 vol% acetonitrile; 30-32 min, 90 vol% acetonitrile; 32–33 min, linear decrease to 10 vol% acetonitrile; 33-35 min, 10 vol% acetonitrile. The product eluted between minutes 20.65 and 23.15. The fractions containing the product were pooled, the organic solvent removed *in vacuo*, and the remaining water layer freeze-dried to obtain the product as a cream-colored solid. Yield: 50 mg (31 µmol, 51%); m.p. > 250 °C (dec.); ^{1}H NMR (400 MHz, 25 °C, DMSO-d_6): δ 9.72 (s, 2H, NH^b), 9.61 (s, 4H, NH^b), 8.47 (d, J = 6.4 Hz, 2H, NH^a), 7.94 (s, 2H, H3), 7.78 – 7.63 (m, 8H, H4'/H5), 7.47 (d, J = 7.4 Hz, 2H, H4), 7.43 (d, J = 7.6 Hz, 2H, H5'), 7.39 (d, J = 7.8 Hz, 4H, H5'), 7.25 (d, J = 8.3 Hz, 2H, H3'), 7.20 (d, J = 8.2 Hz, 2H, H3'), 7.17 (d, J = 8.3

Hz, 2H, H3’), 5.69 (t, J = 7.5 Hz, 2H, Hα’), 5.63 (t, J = 6.6 Hz, 2H, Hα), 5.56 (t, J = 6.9 Hz, 2H, Hα), 4.66 – 4.57 (m, 2H, Hγ’), 3.99 – 3.94 (m, 2H, Hδ’), 3.73 – 3.64 (m, 6H, Hδ/Hδ’), 3.62 – 3.53 (m, 4H, Hδ), 3.15 – 3.05 (m, 8H, H1/H2), 3.00 – 2.91 (m, 2H, Hβ’), 2.59 – 2.50 (m, 4H, Hβ), 2.25 – 2.18 (m, 2H, Hβ’), 2.09 – 1.99 (m, 4H, Hβ), 1.71 – 1.77 (m, 8H, Hγ) ppm; ^{13}C NMR (151 MHz, 25 °C, DMSO-d$_6$): δ 171.0 (C10), 170.9 (C10), 170.8 (C10), 166.9 (C11), 166.0 (C12), 165.9 (C12), 165.8 (C12), 152.0 (C6’), 151.9 (C6’), 151.5 (C6’), 150.5 (C8), 148.6 (C2’), 148.5 (C2’), 148.4 (C2’), 142.4 (C7), 139.1 (C4’), 139.0 (C4’), 138.9 (C4’), 135.1 (C9), 132.8 (C6), 126.4 (C5), 124.7 (C3), 123.1 (C4), 119.8 (C5’), 119.7 (C5’), 116.0 (C3’), 115.8 (C3’), 61.6 (Cα), 61.50 (Cα), 60.6 (Cα’), 52.0 (Cδ‘), 48.2 (Cδ), 46.9 (Cγ‘), 37.1 (Cβ‘), 32.6 (Cβ), 32.5 (Cβ), 31.4 (C1), 30.4 (C2), 22.4 (Cγ), 22.3 (Cγ) ppm; MS (MALDI-TOF, positive mode) *m/z* (%):1602.4 $[M–O+H]^+$ (35), 1617.5 $[M+H^+]$ (54), 1639.5 $[M+Na^+]$ (100), 1655.5 $[M+K^+]$ (37); CHN calculated for $C_{86}H_{80}N_{20}O_{14}\cdot 9\ H_2O$ (M.W.: 1779.82): C, 58.04%; H, 5.55%; N, 15.74%; found: C, 57.92%; H, 5.16%; N, 15.69%.

1.4. NMR and MS Spectra of title compounds

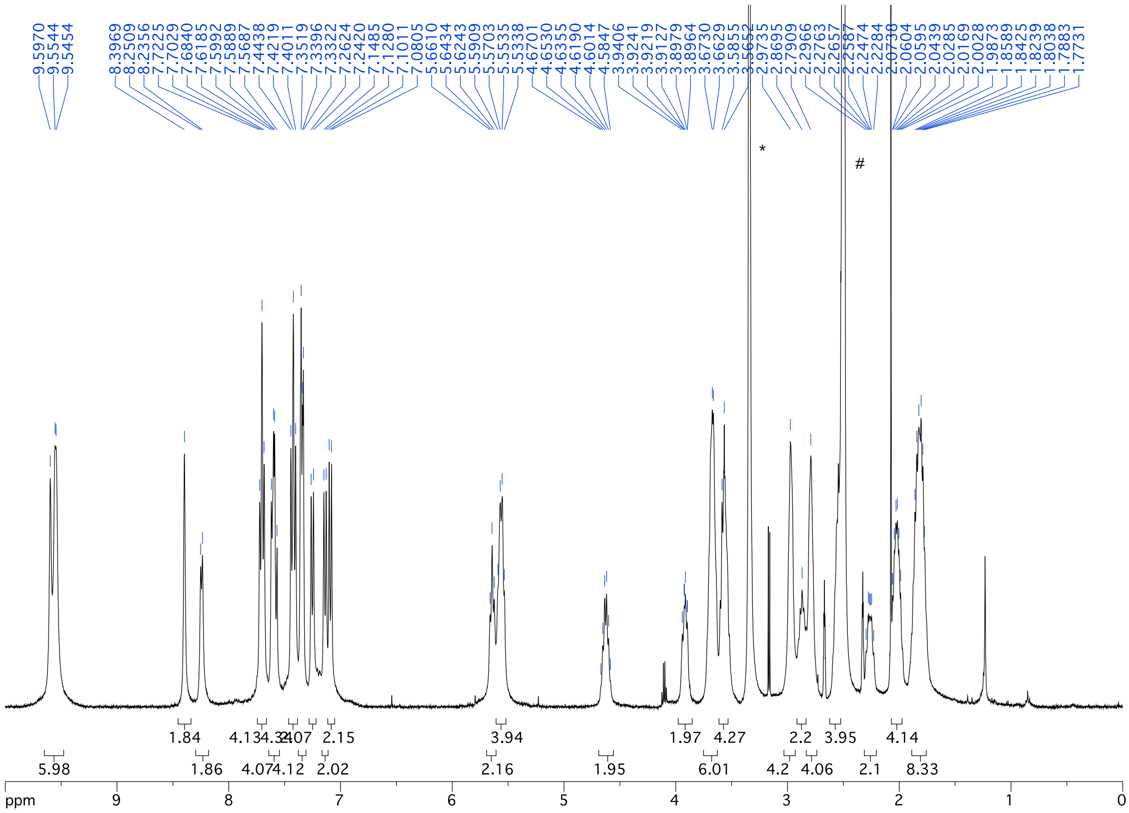

Figure S2. ^{1}H NMR spectrum of (Z)-bis**CP**. Residual solvent peaks: DMSO-d_6 (#), H_2O (*).

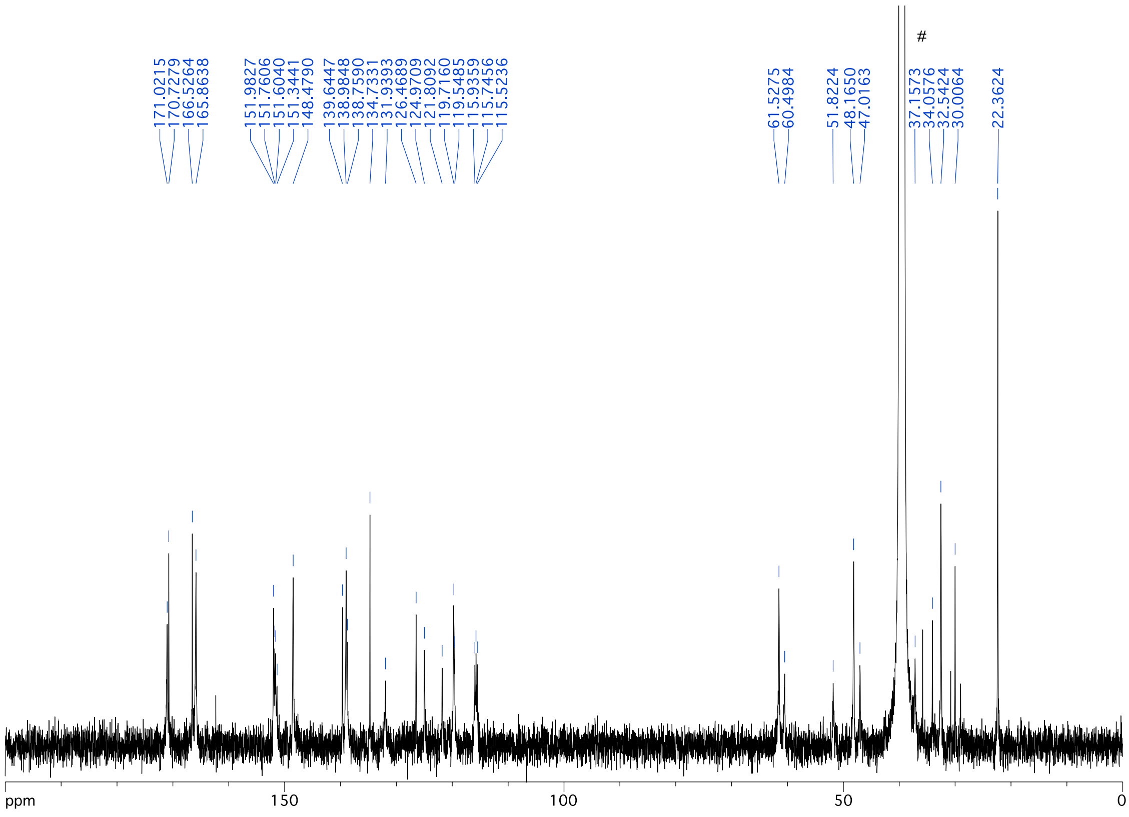

Figure S3. ^{13}C NMR spectrum of (Z)-bis**CP**. Residual solvent peak: DMSO-d_6 (#).

	Int.	*m/z calcd.*	*m/z exp.*
$[M–O+H]^+$	22	1602.69	1602.60
$[M+H]^+$	35	1617.62	1617.68
$[M+Na]^+$	100	1639.61	1639.65
$[M+K]^+$	29	1655.58	1655.70

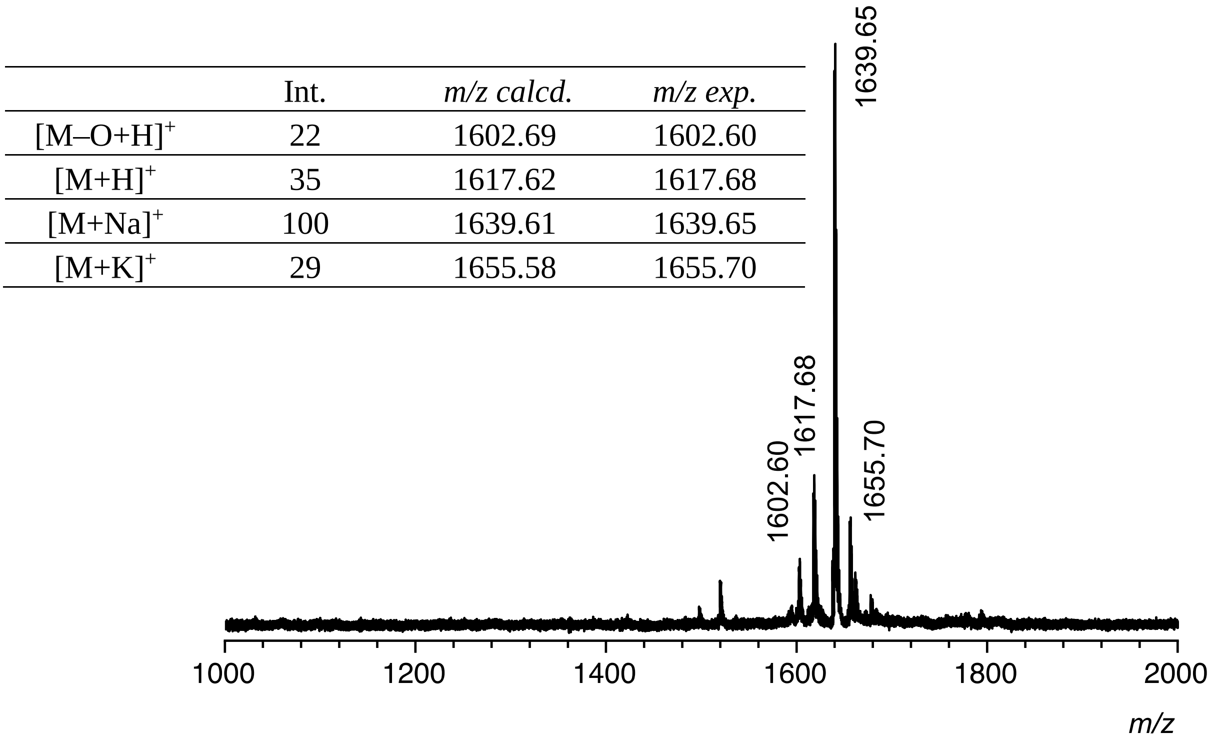

Figure S4. MALDI-MS spectrum of (*Z*)-bis**CP** with peak assignment.

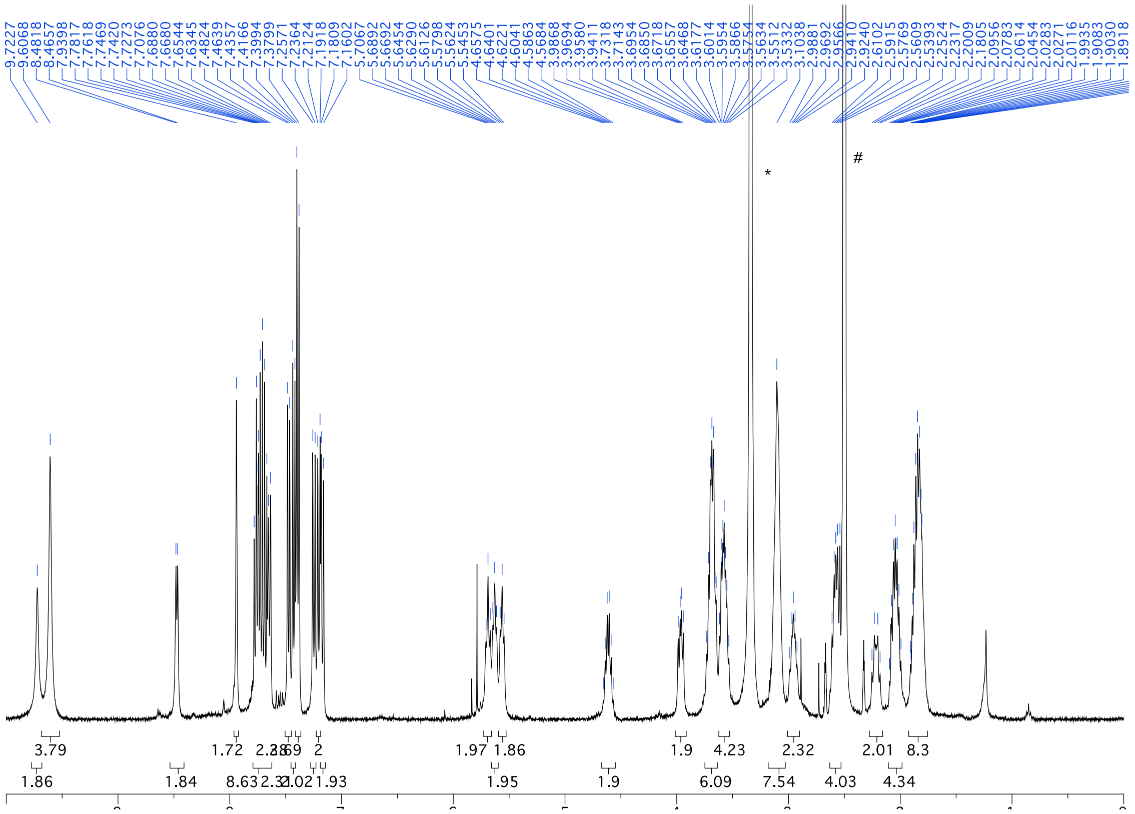

Figure S5. ^1H NMR spectrum of (*E*)-bis**CP**. Residual solvent peaks: DMSO-d_6 (#), H_2O (*).

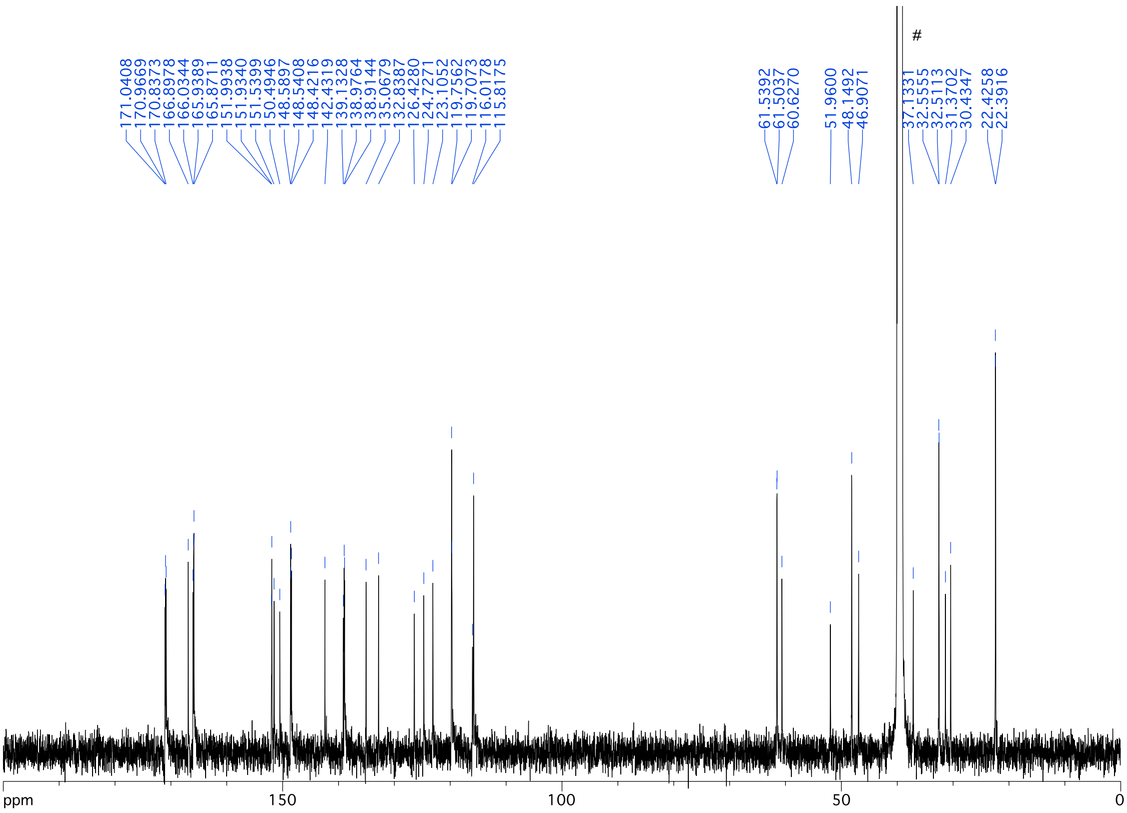

Figure S6. ^{13}C NMR spectrum of (*E*)-bis**CP**. Residual solvent peak: DMSO-d_6 (#).

	Int.	*m/z calcd.*	*m/z exp.*
[M–O+H]$^+$	35	1602.69	1602.44
[M+H]$^+$	54	1617.62	1617.52
[M+Na]$^+$	100	1639.61	1639.51
[M+K]$^+$	37	1655.58	1655.53

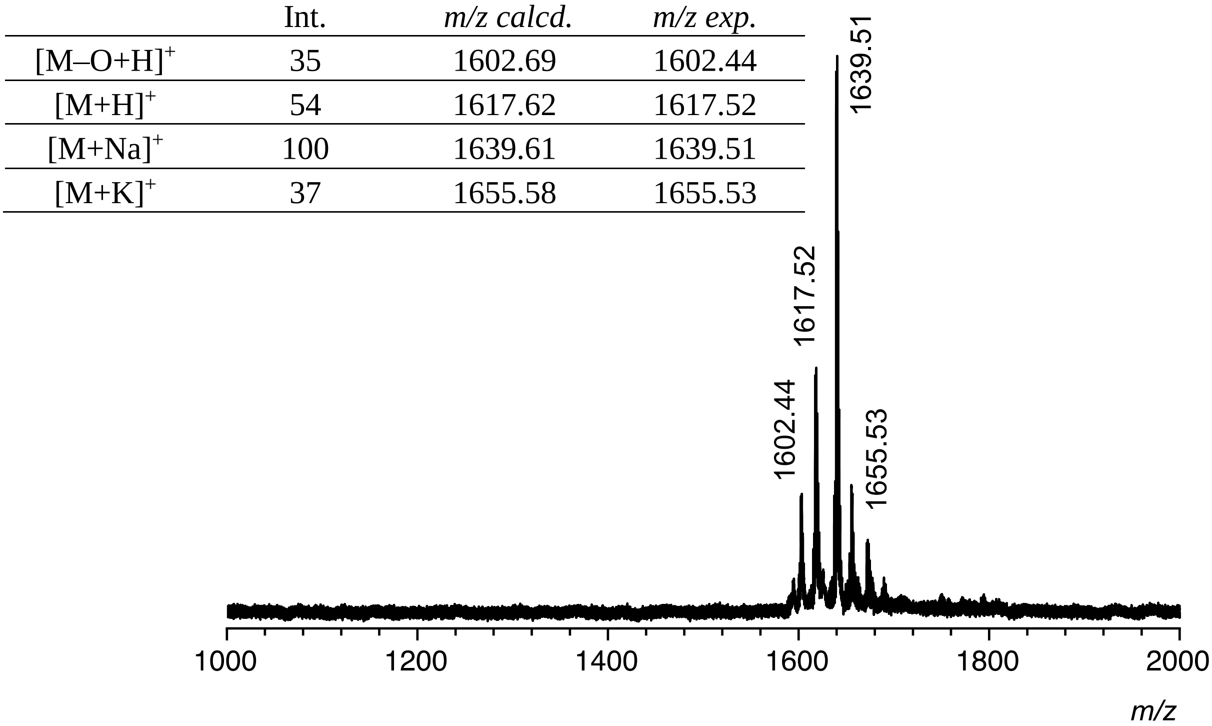

Figure S7. MALDI-MS spectrum of (*E*)-bis**CP** with peak assignment.

2. ^{1}H NMR spectroscopic titrations

2.1. Titration with receptor (*E*/*Z*)-bis**CP**

For ^{1}H NMR titrations using (*E*)- and (*Z*)-bis**CP**, 0.5 mM solutions in a mixture of 0.5 vol% H_2O/DMSO-d_6 were prepared. Then, 5 mM solutions of the tetrabutylammonium anion were prepared using these solutions to prevent dilution of the receptor during the titration. The 5 mM anion solution was added stepwise to 0.5 mL of the receptor solution and a ^{1}H NMR spectrum (500 MHz, 298 K) was recorded after each addition. When a fast-exchanging complex was observed, the addition steps constituted volumes of 10 × 10 μL, 5 × 20 μL, 2 × 50 μL and 2 × 100 μL adding up to a total volume of 500 mL guest solution. In case of a slow-exchanging complex, the added volumes were adjusted to contain 0.1 eq of the guest. The solutions of TBA_2SO_4 in particular suffered from proton exchange due to their increased basicity and the presence of water in the host and guest solutions. To ensure that all guest added to the host solutions existed as dianionic SO_4^{2-} species, a small amount of DIPEA (1 equiv. with respect to the total amount of host in solution) was added to the host solution prior to the start of the titration. Data was fitted using HypNMR.[4]

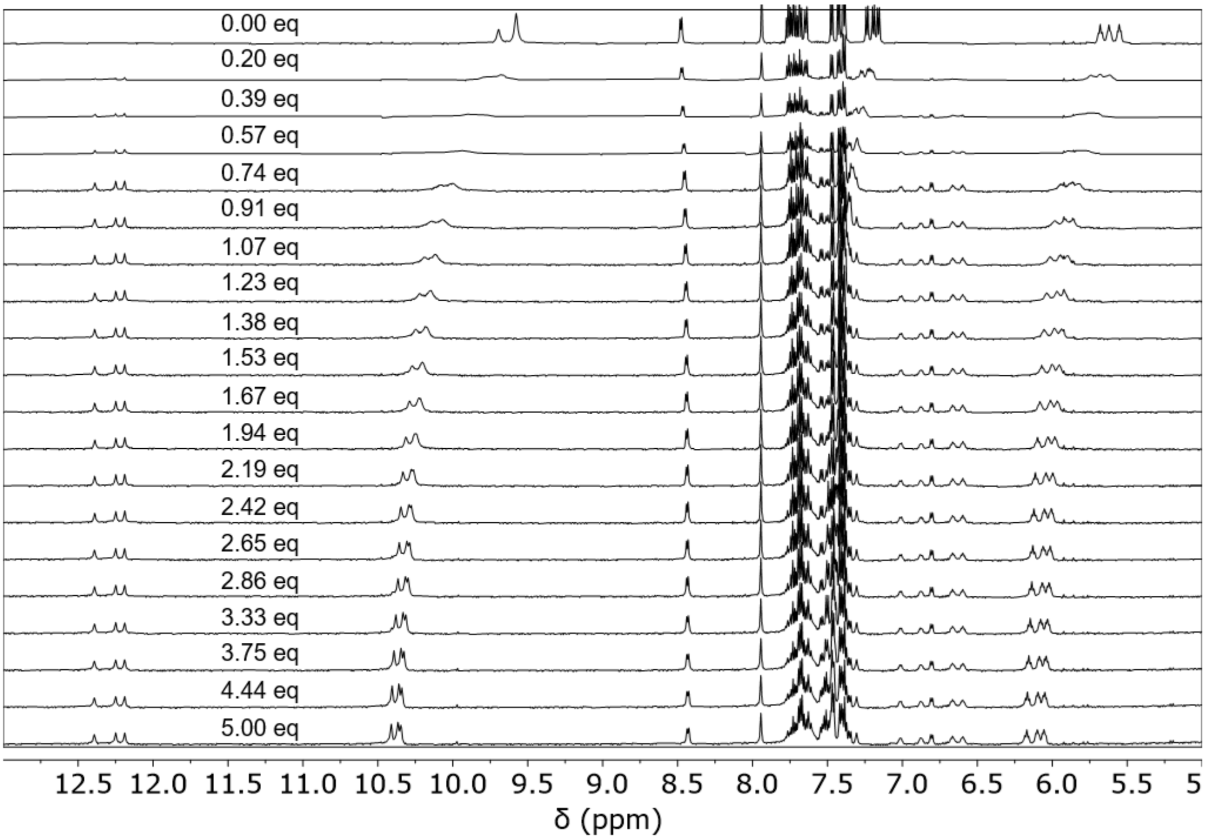

Figure S8. ^{1}H NMR spectral changes of (*E*)-bis**CP** in 0.5 vol% H_2O/DMSO-d_6 (0.5 mM) upon the stepwise addition of TBA_2SO_4.

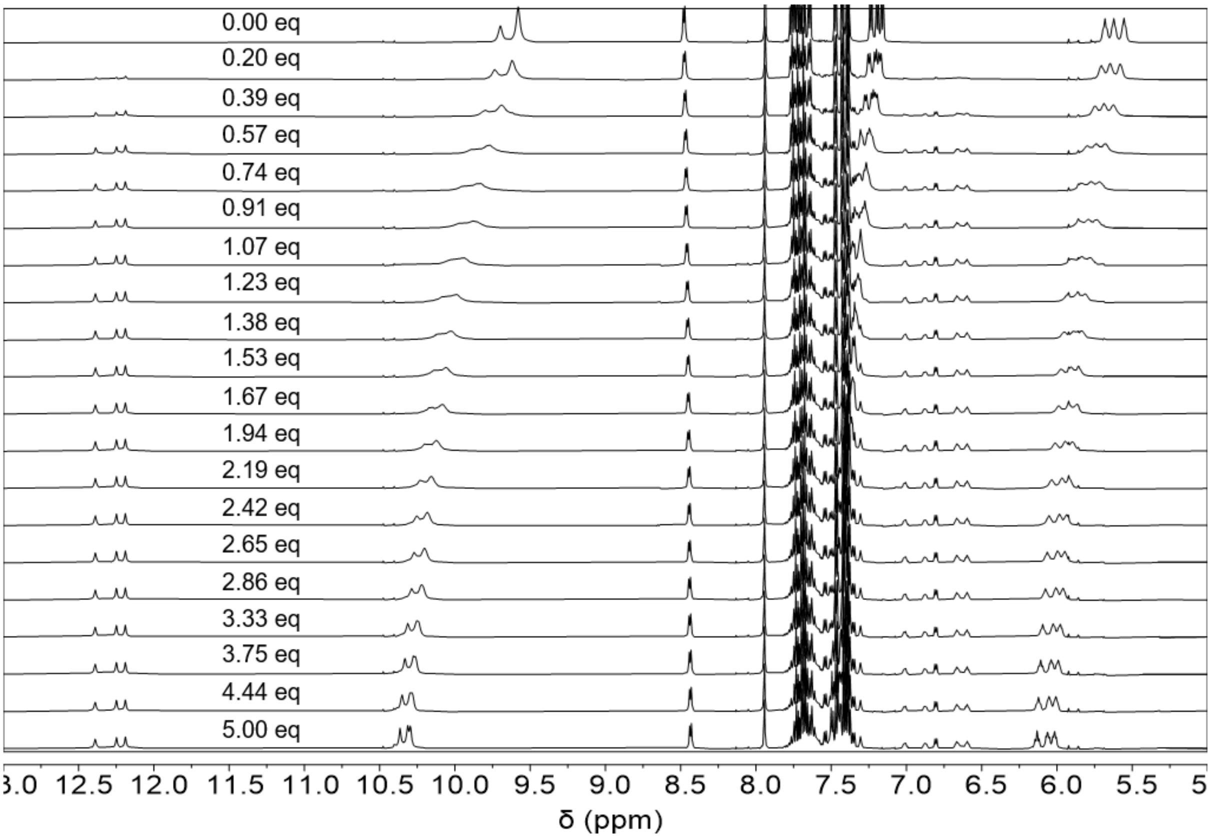

Figure S9. ^{1}H NMR spectral changes of (*E*)-bis**CP** in 0.5 vol% H_2O/DMSO-d_6 (0.5 mM) upon the stepwise addition of $TBAHSO_4$.

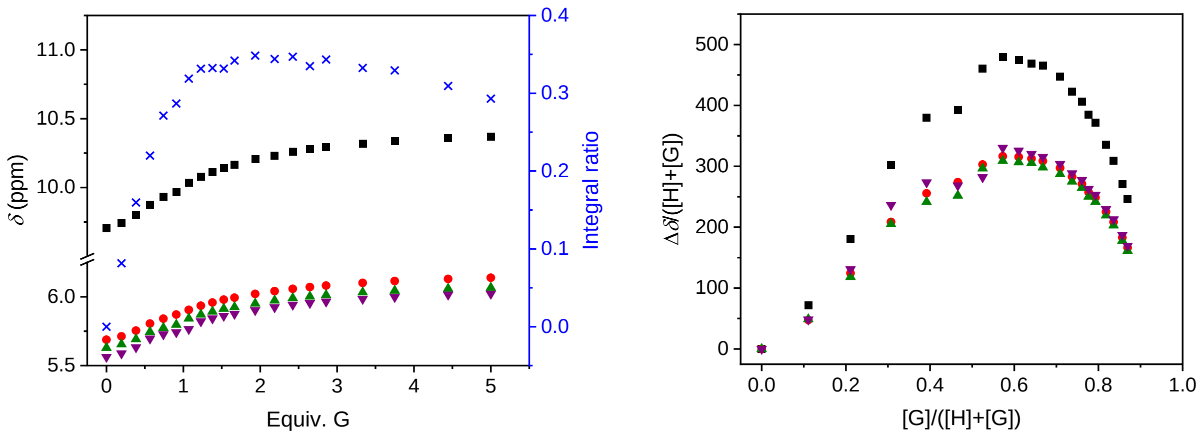

Figure S10. (left) Titration curves for the gradually shifted NH (black) and H(α) (red, green, purple) proton signals upon the addition of $TBAHSO_4$ (G) to (*E*)-bis**CP** (H) and relative integral ratio (blue) of signals in the slow- and fast-exchanging regime. (right) Corresponding modified Job plot constructed using the gradually shifted signals and concentrations of remaining sulfate (G) and (*E*)-bis**CP** (H) not forming the slow-exchanging 2:2 H-G species, calculated using the integral ratios.

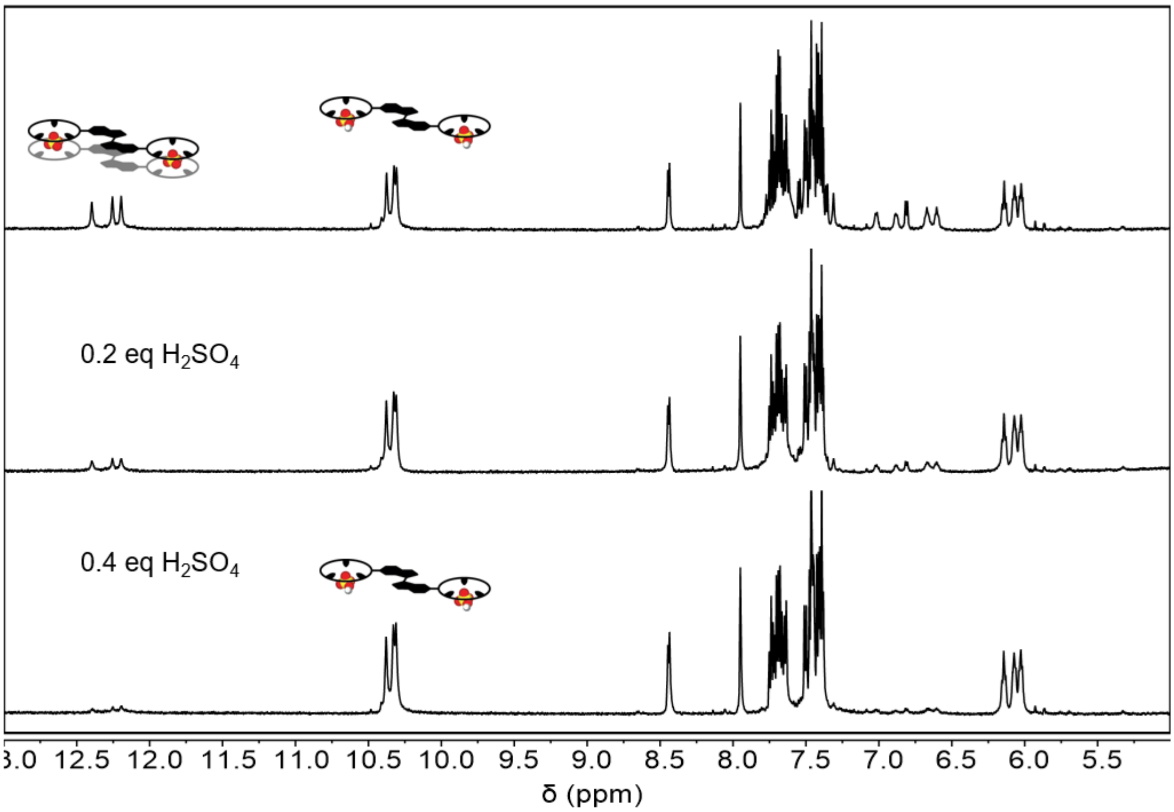

Figure S11. ^{1}H NMR spectral changes of a mixture of (*E*)-bis**CP**·$(HSO_4^-)_2$ and [(*E*)-bis**CP**·$SO_4^{2-}]_2$ in 0.5 vol% H_2O/DMSO-d_6 (0.5 mM) upon the stepwise addition of H_2SO_4 (50 mM) to form exclusively (*E*)-bis**CP**·HSO_4^-.

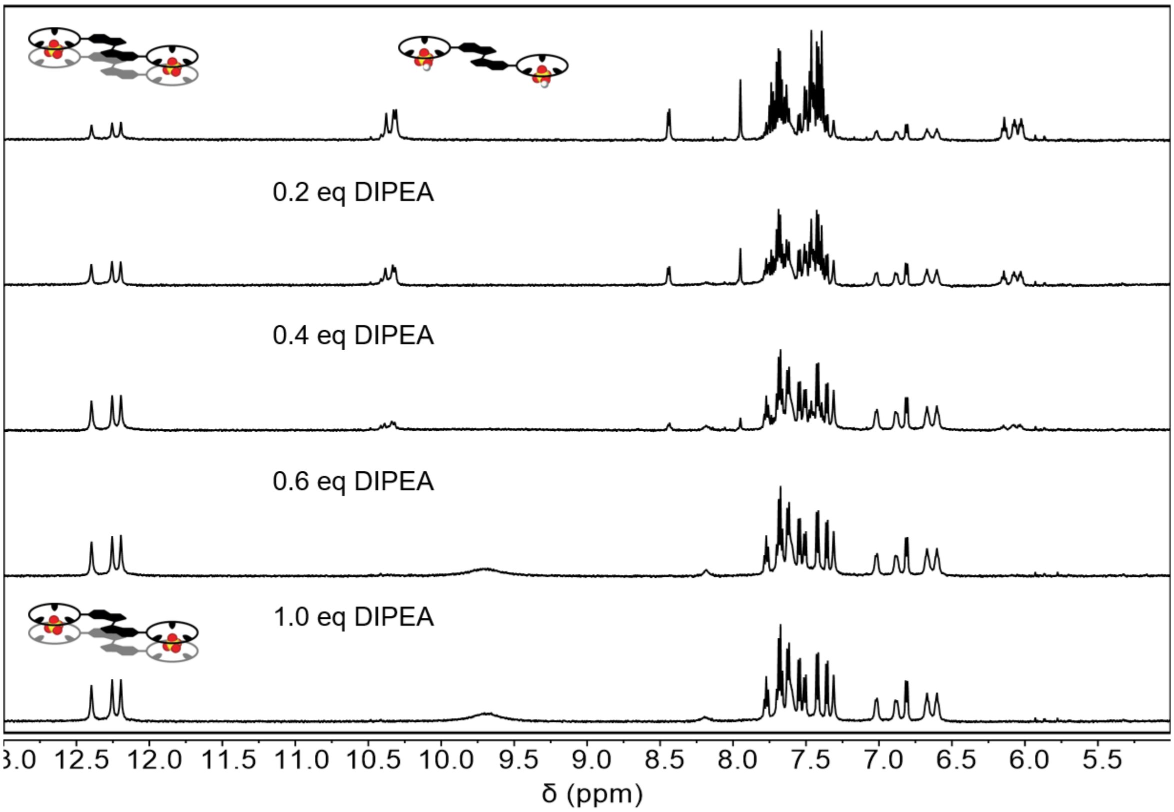

Figure S12. ^{1}H NMR spectral changes of a mixture of (*E*)-bis**CP**·$(HSO_4^-)_2$ and [(*E*)-bis**CP**·$SO_4^{2-}]_2$ in 0.5 vol% H_2O/DMSO-d_6 (0.5 mM) upon the stepwise addition of DIPEA to form exclusively [(*E*)-bis**CP**·$SO_4^{2-}]_2$.

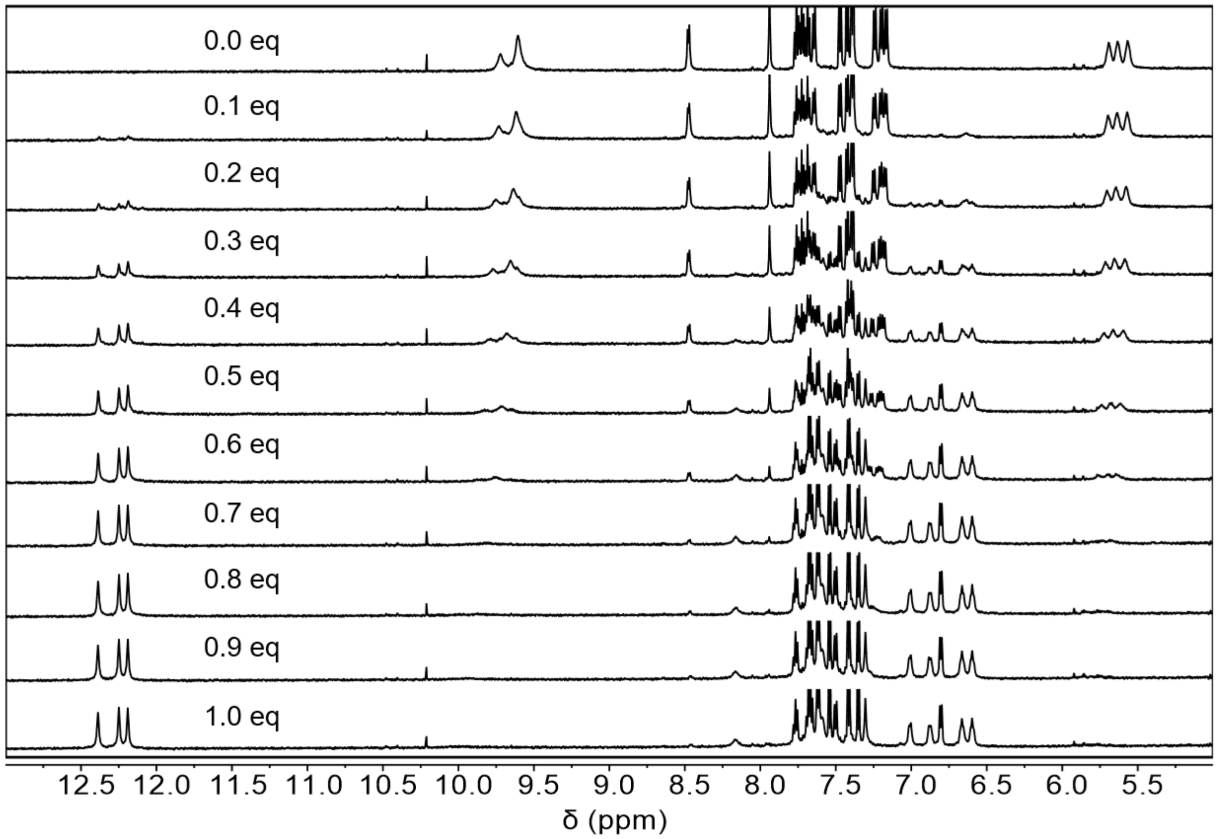

Figure S13. ^{1}H NMR spectral changes of (*E*)-bis**CP** in 0.5 vol% H_2O/0.05 vol% DIPEA/DMSO-d_6 (0.5 mM) upon the stepwise addition of TBA_2SO_4.

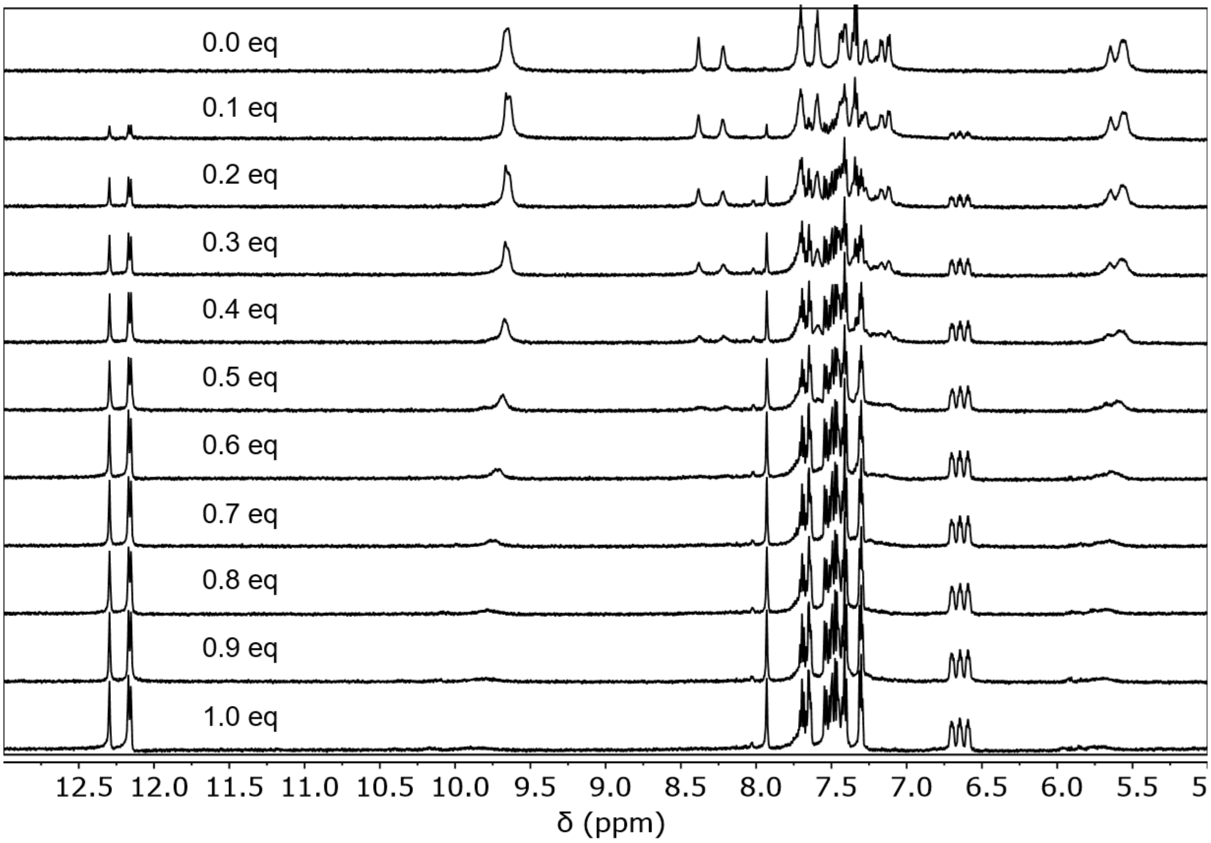

Figure S14. ^{1}H NMR spectral changes of (*Z*)-bis**CP** in 0.5 vol% H_2O/DMSO-d_6 (0.5 mM) upon the stepwise addition of $TBAHSO_4$.

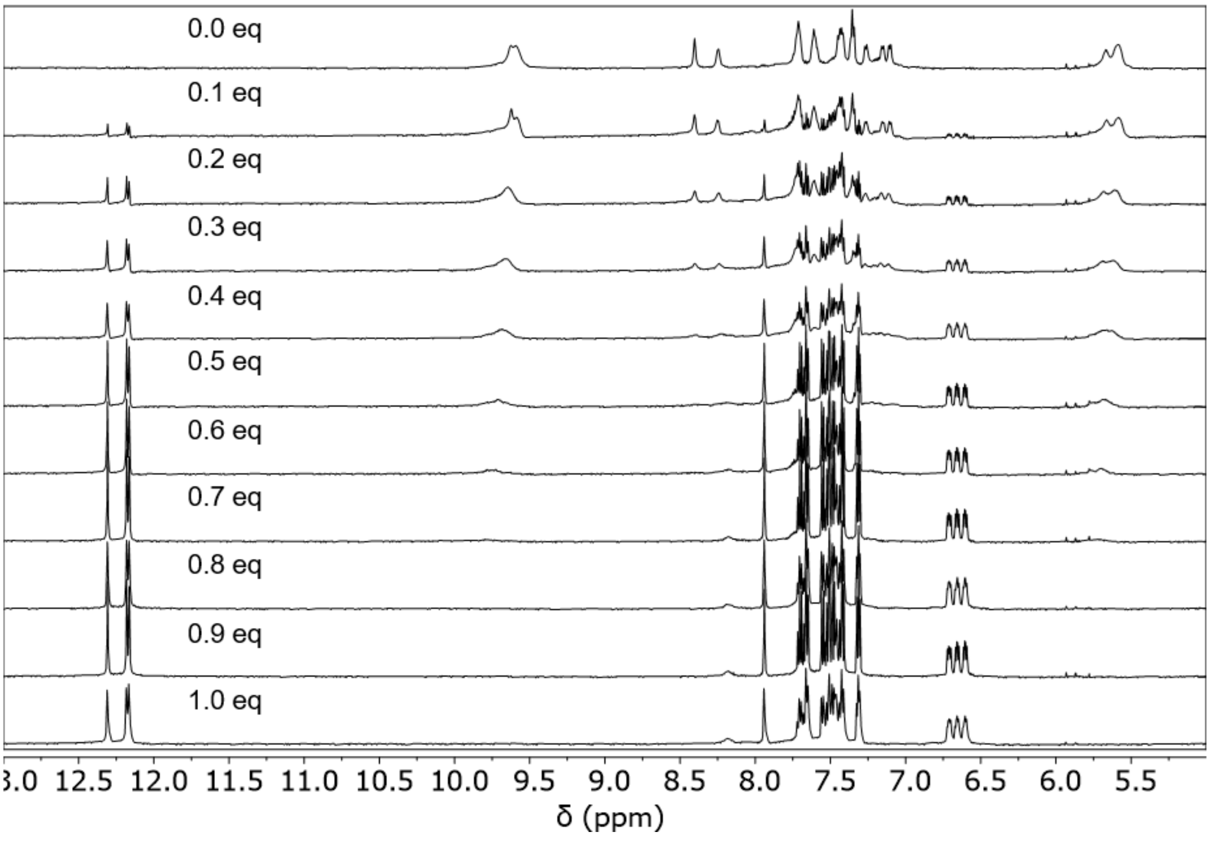

Figure S15. ^{1}H NMR spectral changes of (*Z*)-bis**CP** in 0.5 vol% H_2O/0.05 vol% DIPEA/DMSO-d_6 (0.5 mM) upon the stepwise addition of TBA_2SO_4.

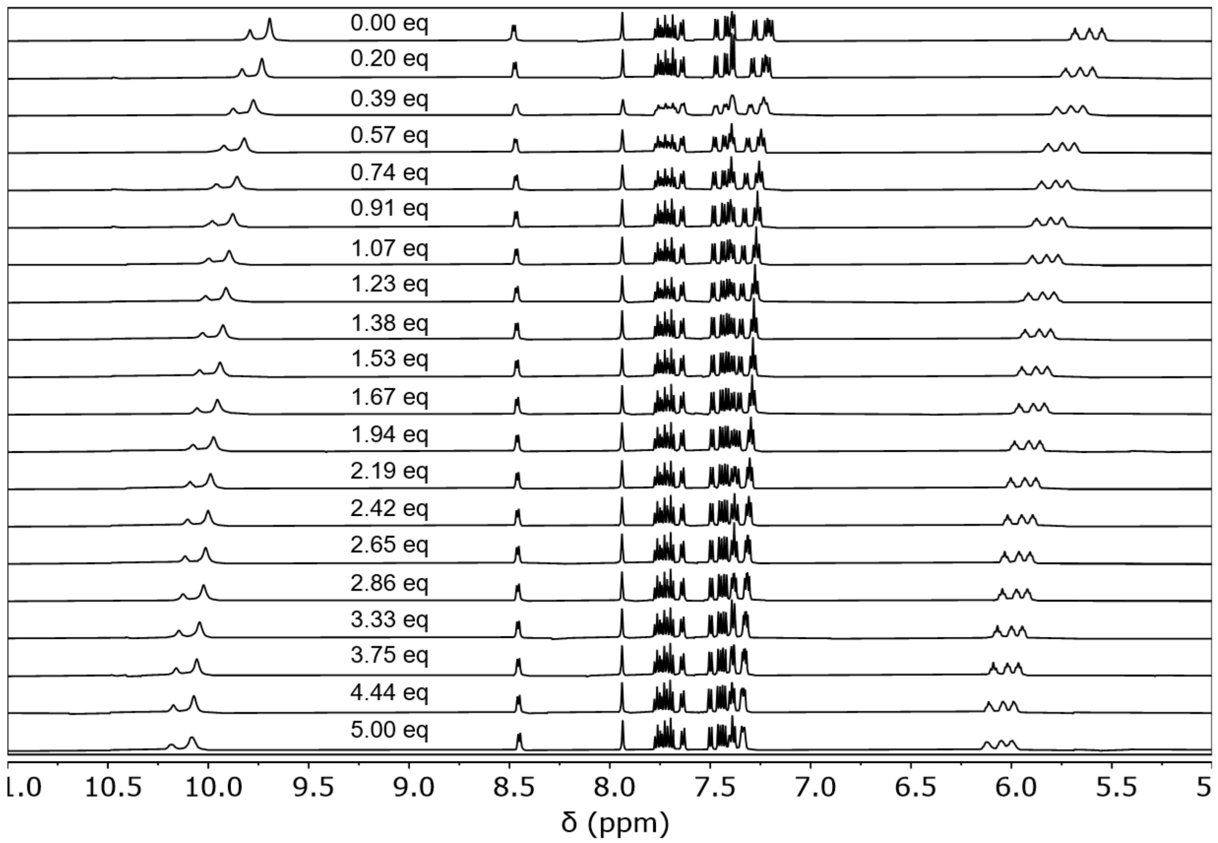

Figure S16. ^{1}H NMR spectral changes of (*E*)-bis**CP** in 0.5 vol% H_2O/DMSO-d_6 (0.5 mM) upon the stepwise addition of TBABr.

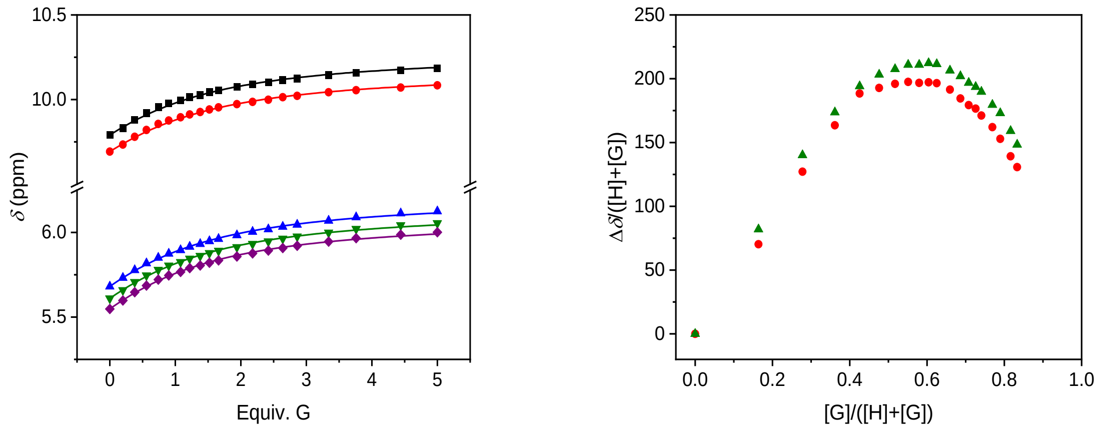

Figure S17. (left)Titration curves for the NH (black, red) and H(α) (blue, green, purple) proton signals generated from addition of TBABr to (*E*)-bis**CP** and data fitting obtained using a 1:2 model assuming $\alpha = 1$ ($K_{11} = 2.0 \times 10^3$ M^{-1}, $K_{12} = 1.0 \times 10^6$ M^{-1}). (right) Corresponding modified Job plot. For a description of the data fitting procedure, see reference [5].

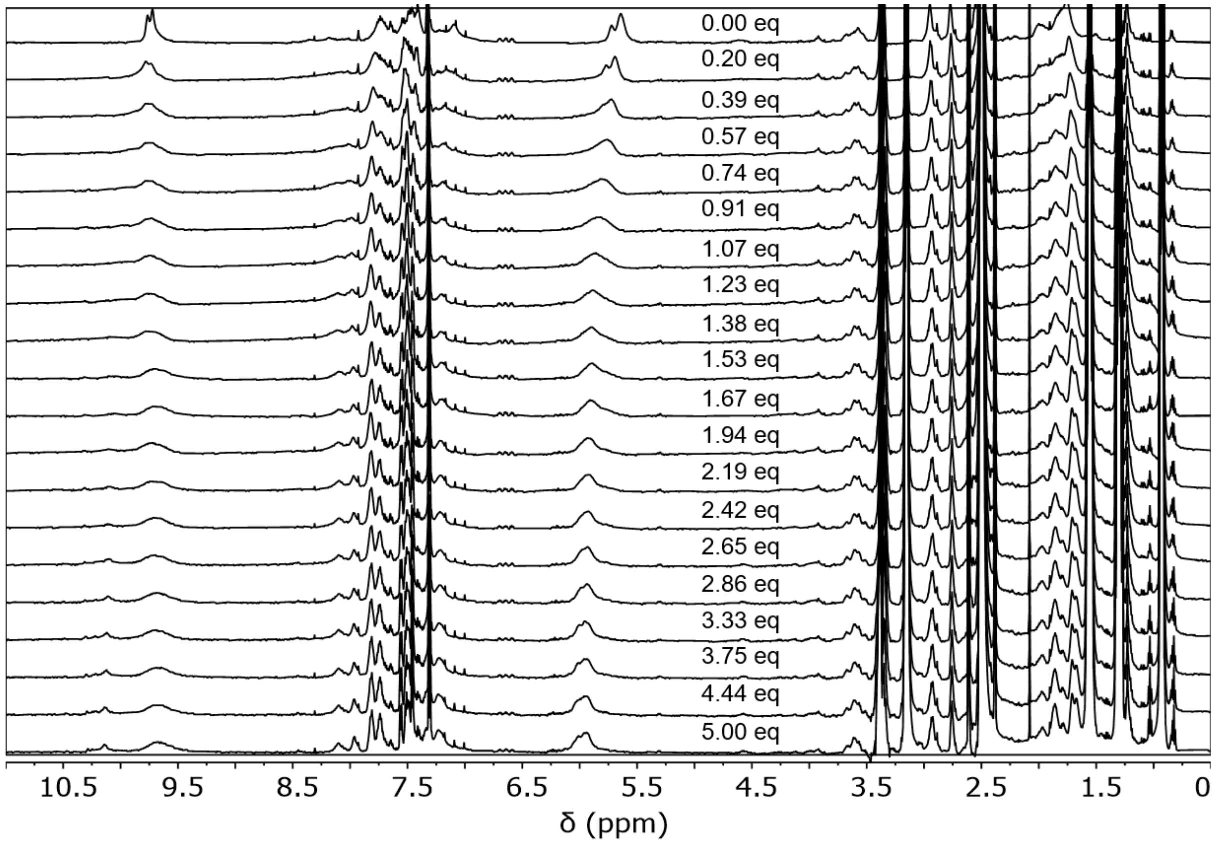

Figure S18. ^{1}H NMR spectral changes of (*Z*)-bis**CP** in 0.5 vol% H_2O/DMSO-d_6 (0.5 mM) upon the stepwise addition of TBABr.

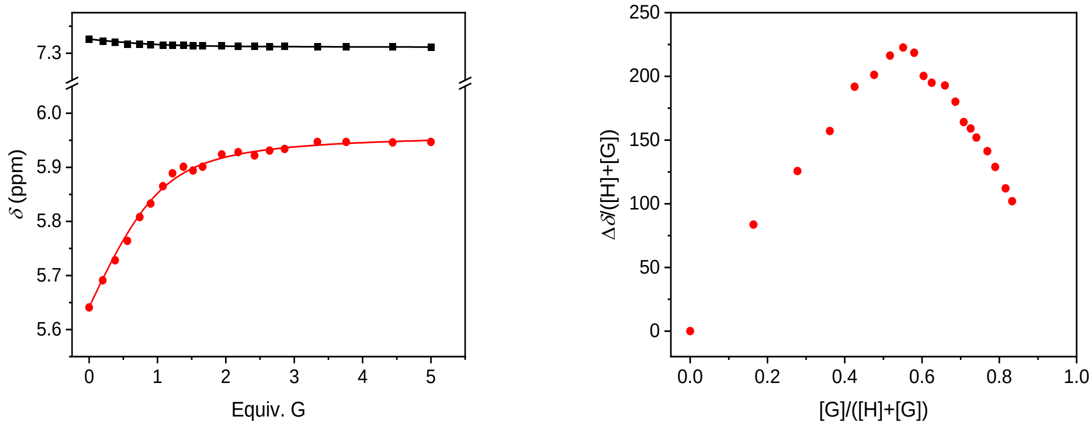

Figure S19. (left) Titration curves for the NH (black) and H(α) (red) proton signals generated from addition of TBABr (G) to (*Z*)-bis**CP** (H) fitted and data fitting obtained using a 1:1 binding model (K_a = 1.1 × 10^4 M^{-1}). (right) Corresponding modified Job plot.

2.2. Titration with model compound **CP**

For ^{1}H NMR titrations of **CP**, a 1.0 mM solution in a mixture of 0.5 vol% H_2O/DMSO-d_6 was prepared . Then, 10 mM solutions of the tetrabutylammonium salt were prepared using this **CP** solution (1.0 mM) to prevent dilution of the receptor during the titration. The 10 mM salt solution was added stepwise to 0.5 mL of the receptor solution and a ^{1}H NMR spectrum (500 MHz, 298 K) was recorded after each addition. The addition steps constituted volumes of 10 × 10 µL, 5 × 20 µL, 2 × 50 µL and 2 × 100 µL adding up to a total volume of 500 mL guest solution added. Data was fitted using HypNMR.[4]

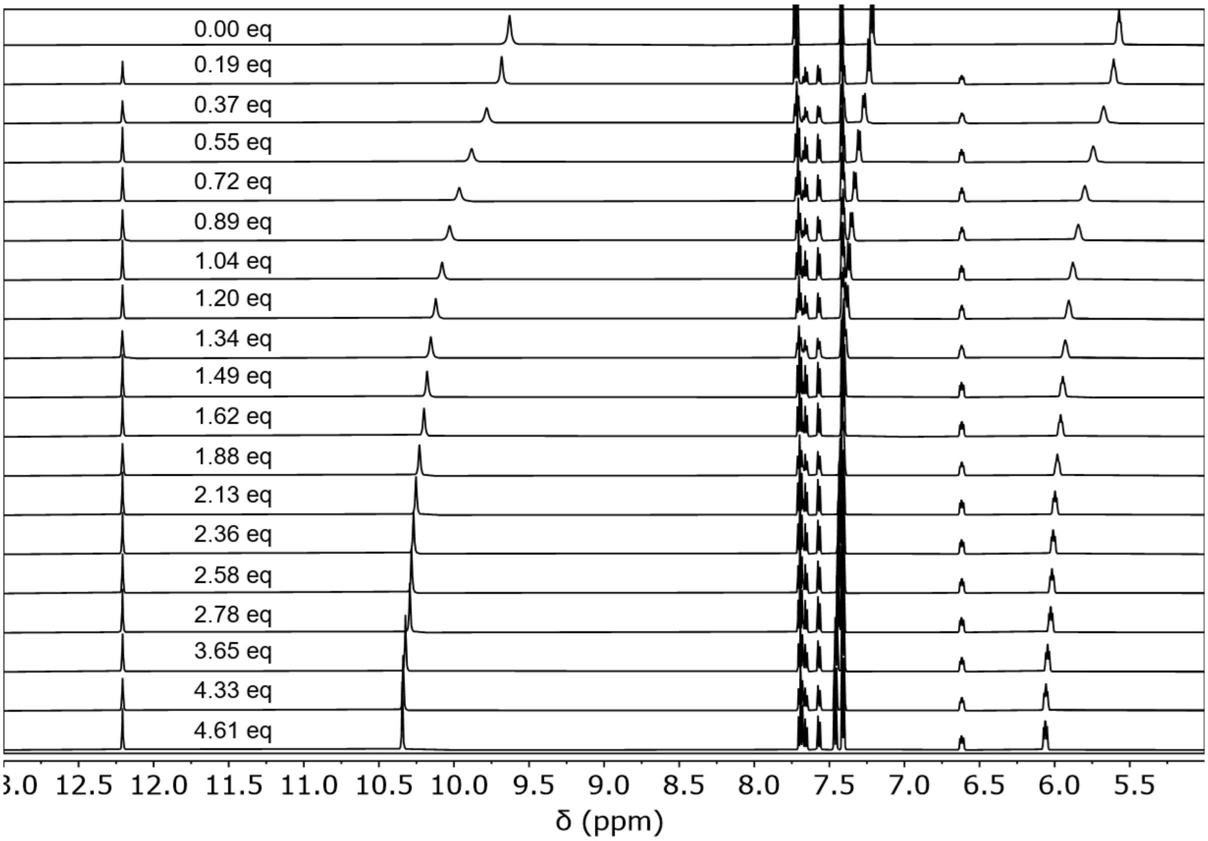

Figure S20. ^{1}H NMR spectral changes of **CP** (0.5 vol% H_2O/DMSO-d_6) upon the stepwise addition of $TBAHSO_4$.

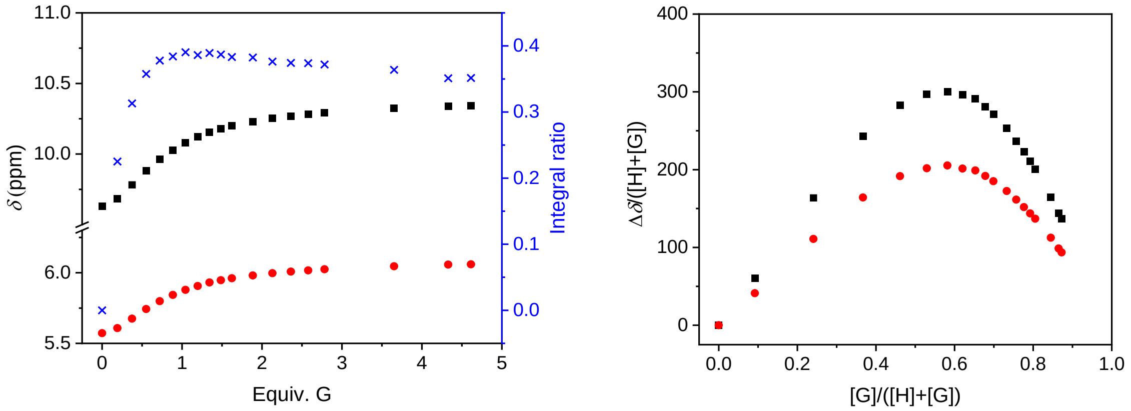

Figure S21. (left) Titration curves for the gradually shifted NH (black) and H(α) (red) proton signals upon the addition of $TBAHSO_4$ (G) to **CP** (H) and relative integral ratio (blue) of signals in the slow- and fast-exchanging regime. (right) Corresponding modified Job plot constructed using the gradually shifted signals and concentrations of remaining sulfate (G) and **CP** (H) not forming the slow-exchanging 2:1 H-G species, calculated using the integral ratios.

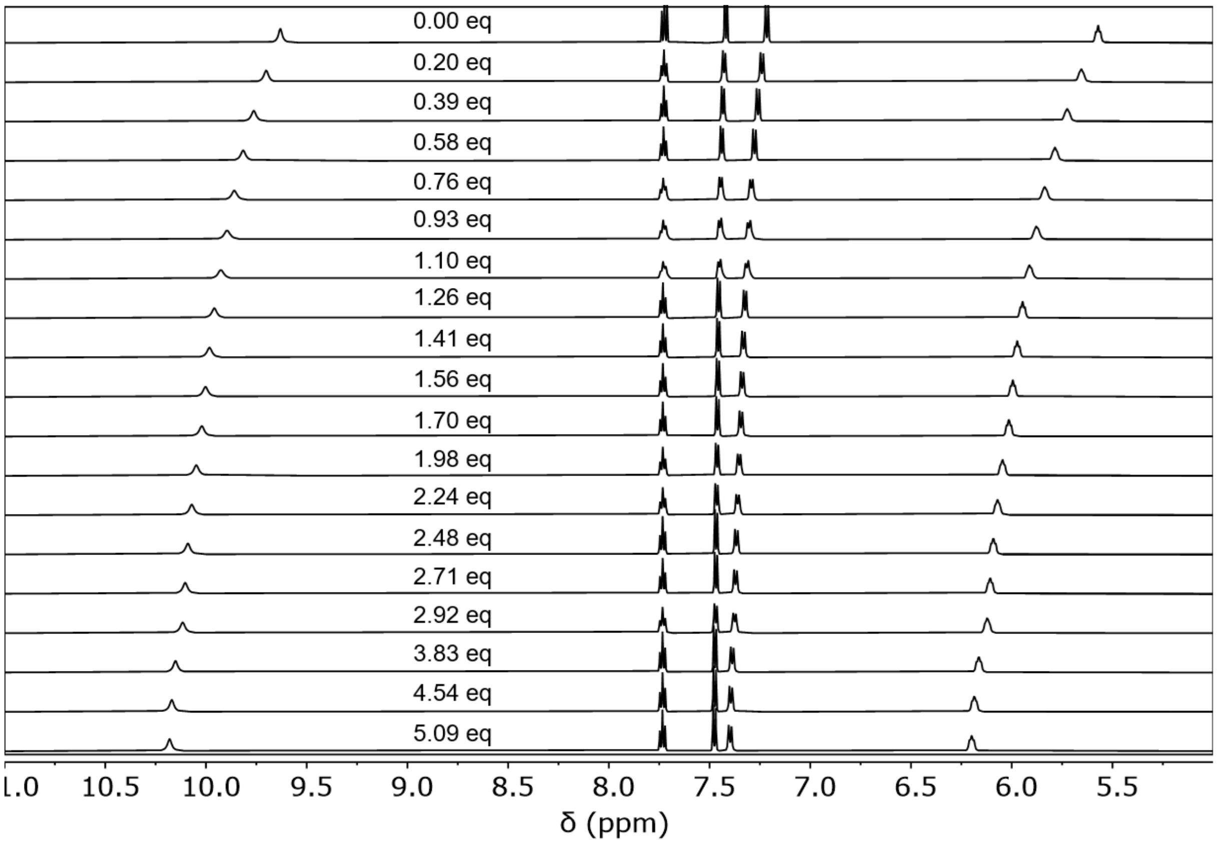

Figure S22. ^{1}H NMR spectral changes of **CP** (0.5 vol% H_2O/DMSO-d_6) upon the stepwise addition of TBABr.

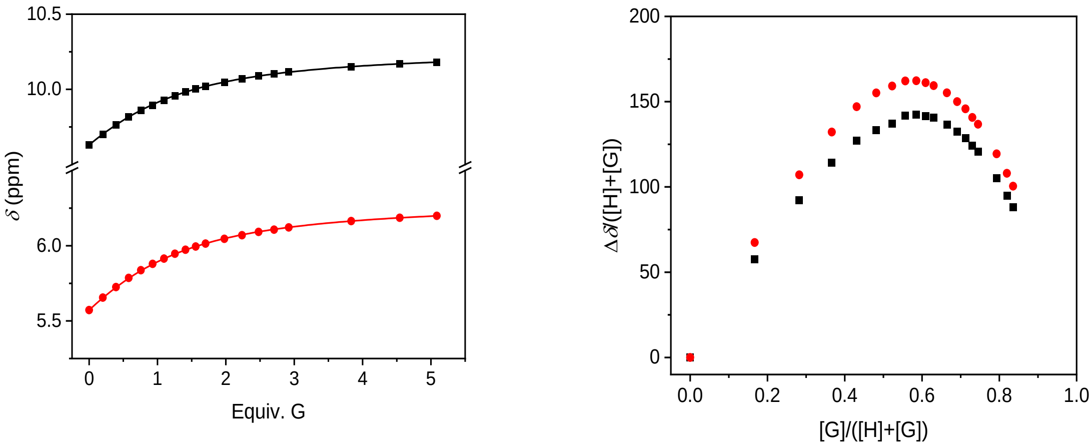

Figure S23. (left) Titration curve for the NH (black) and H(α) (red) proton signals generated from the addition of TBABr (G) to **CP** (H) and data fitting obtained using a 1:1 binding model (K_a = 1.4 × 10^3 M^{-1}). (right) Corresponding modified Job plot.

3. Mass spectrometric binding studies

Equal volumes (0.2 mL) of a solution of (*E*)- or (*Z*)-bis**CP** in methanol (2 mM) and a solution of Na_2SO_4 in water (2 mM) were mixed. The mixture was diluted with methanol (3.6 mL), to give a solution that was 0.1 mM in both the corresponding bis(cyclopeptide) and SO_4^{2-}. This solution was immediately subjected to the ESI-MS measurement.

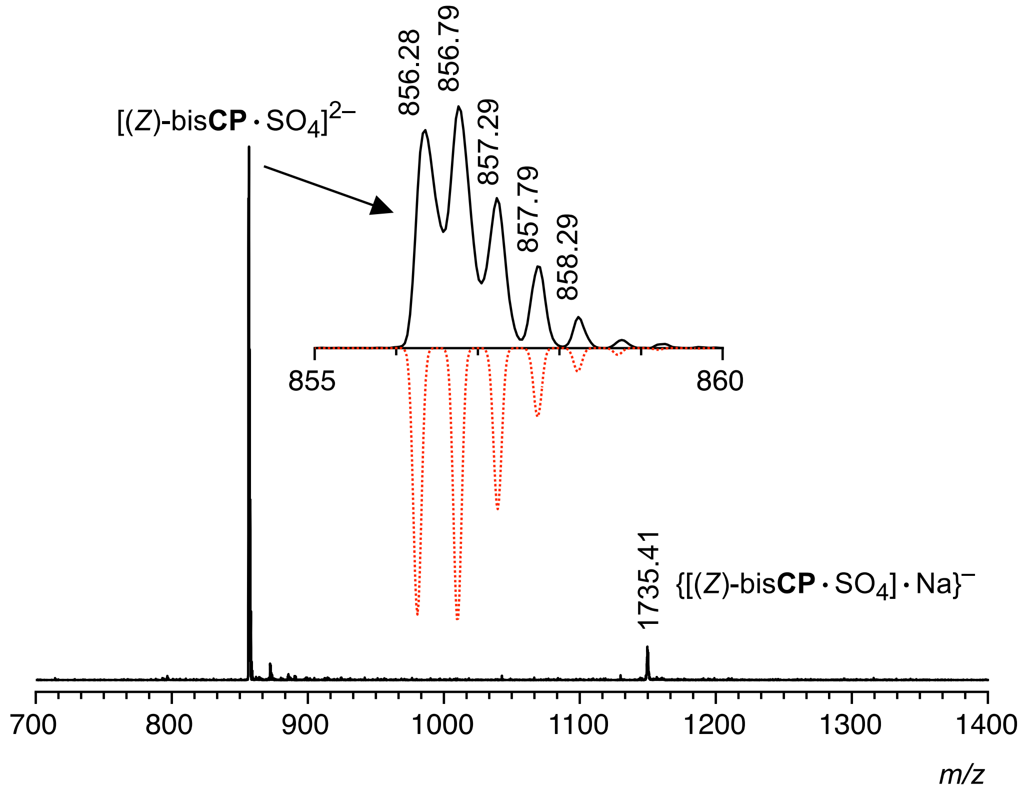

Figure S24. ESI-MS spectrum of (*Z*)-bis**CP** in 5 vol% water/methanol in the presence of Na_2SO_4. The inset shows the experimental and calculated isotope pattern for an ion with the composition [(*Z*)-bis**CP**·SO_4]$^{2-}$.

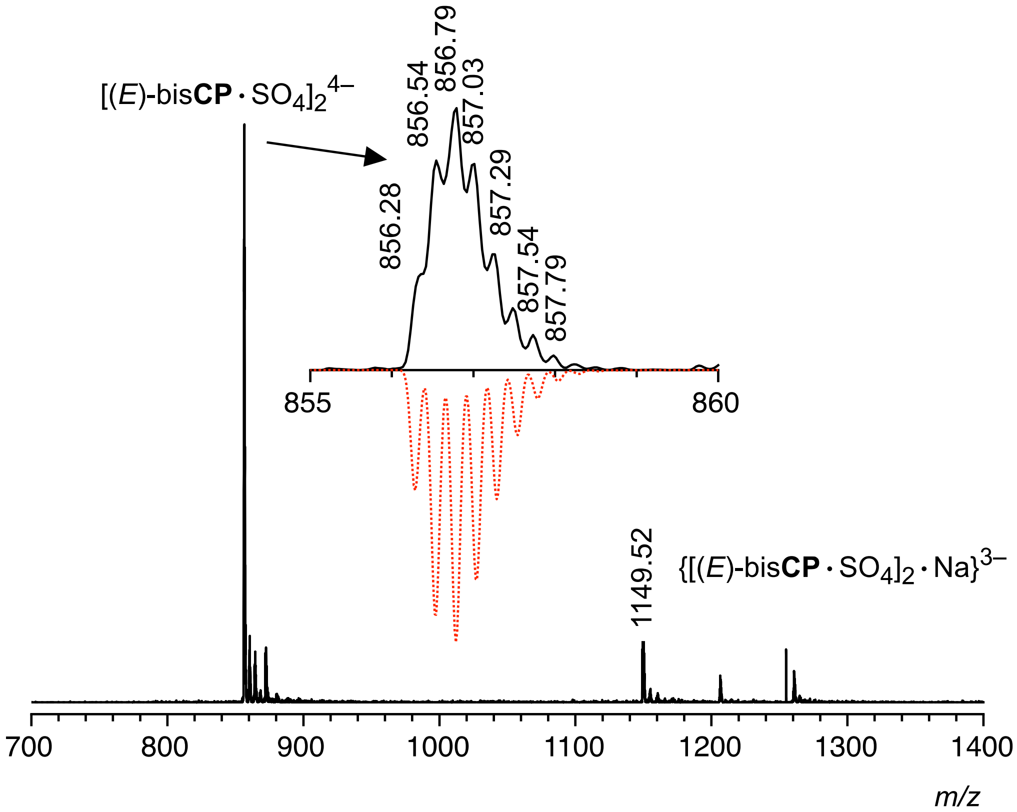

Figure S25. ESI-MS spectrum of (*E*)-bis**CP** in 5 vol% water/methanol in the presence of Na_2SO_4. The inset shows the experimental and calculated isotope pattern for an ion with the composition [(*E*)-bis**CP**·SO_4^{2-}]$_2$.

4. UV-vis photoisomerisation studies

A 10 μM solution of the (*E*)-bis**CP** in 0.5 vol% H_2O/DMSO was prepared. Of this stock solution, 2.5 mL was transferred to a quartz cuvette (1 cm pathlength) and the solution was degassed with argon for 2 min prior to being sealed with a Teflon stopper.

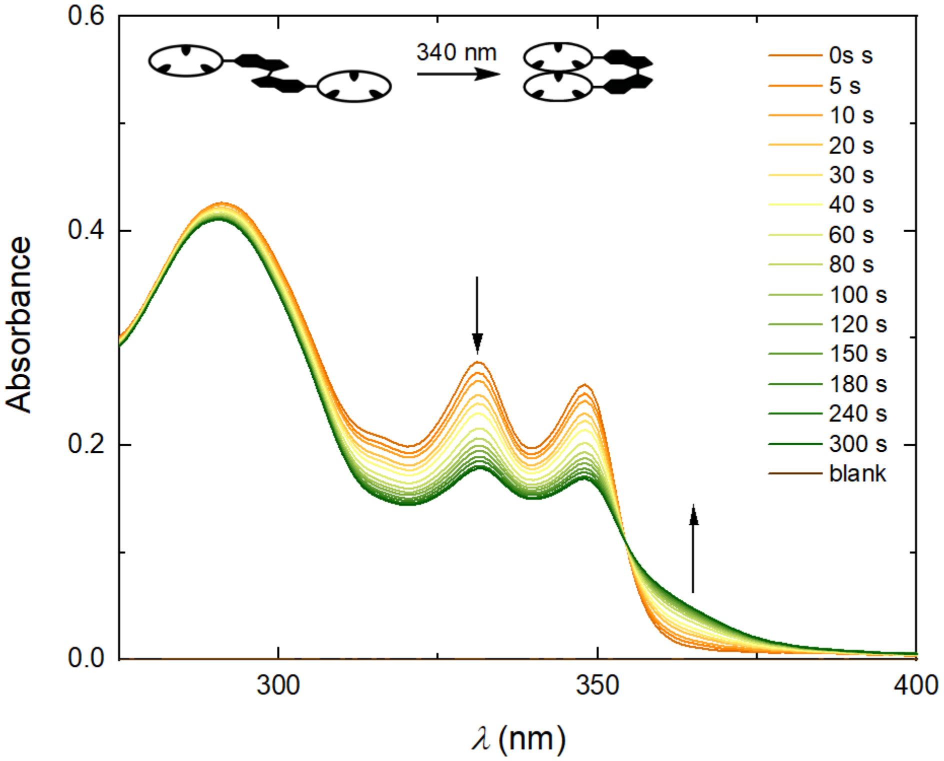

Figure S26. UV-vis spectra of (*E*)-bis**CP** in degassed 0.5 vol% H_2O/DMSO (10 μM) before and after irradiation with 340 nm showing an isosbestic point at *λ* = 355 nm.

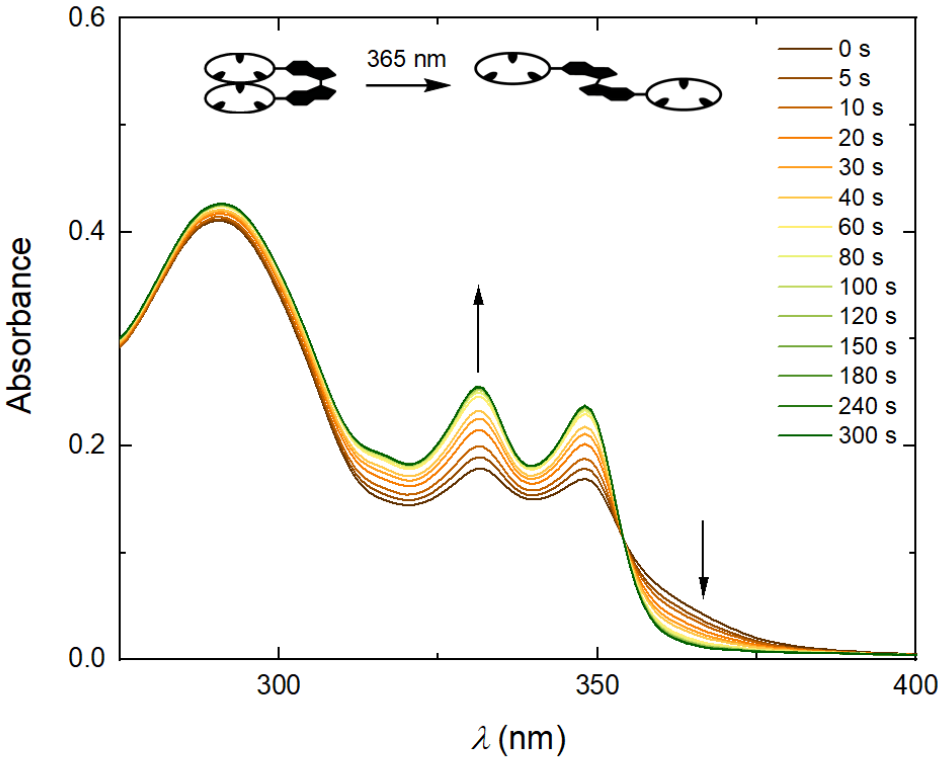

Figure S27. UV-vis spectra of (Z)-bis**CP** in degassed 0.5 vol% H2O/DMSO (10 μM) before and after irradiation with 365 nm showing an isosbestic point at λ = 355 nm.

5. ¹H NMR photoisomerisation studies

5.1. Sample irradiation at room temperature

For the photoisomerisation studies using ¹H NMR spectroscopy, solutions of (*E*)- and (*Z*)-bis**CP** were prepared in 0.5 vol% H_2O/DMSO at a concentration of 0.5 mM. In selected cases either 0.4 equiv. H_2SO_4 or 0.05 vol% DIPEA was present to ensure full protonation and complete deprotonation of the sulfate anion, respectively. For the salt-containing samples, the respective anion was added in 10-fold excess (5 mM). Samples were irradiated using 340 nm and 365 nm LEDs for varying times. For determination of the PSS ratios, the NH signals in the aromatic region of the (*E*)- and (*Z*)-isomer were integrated and compared.

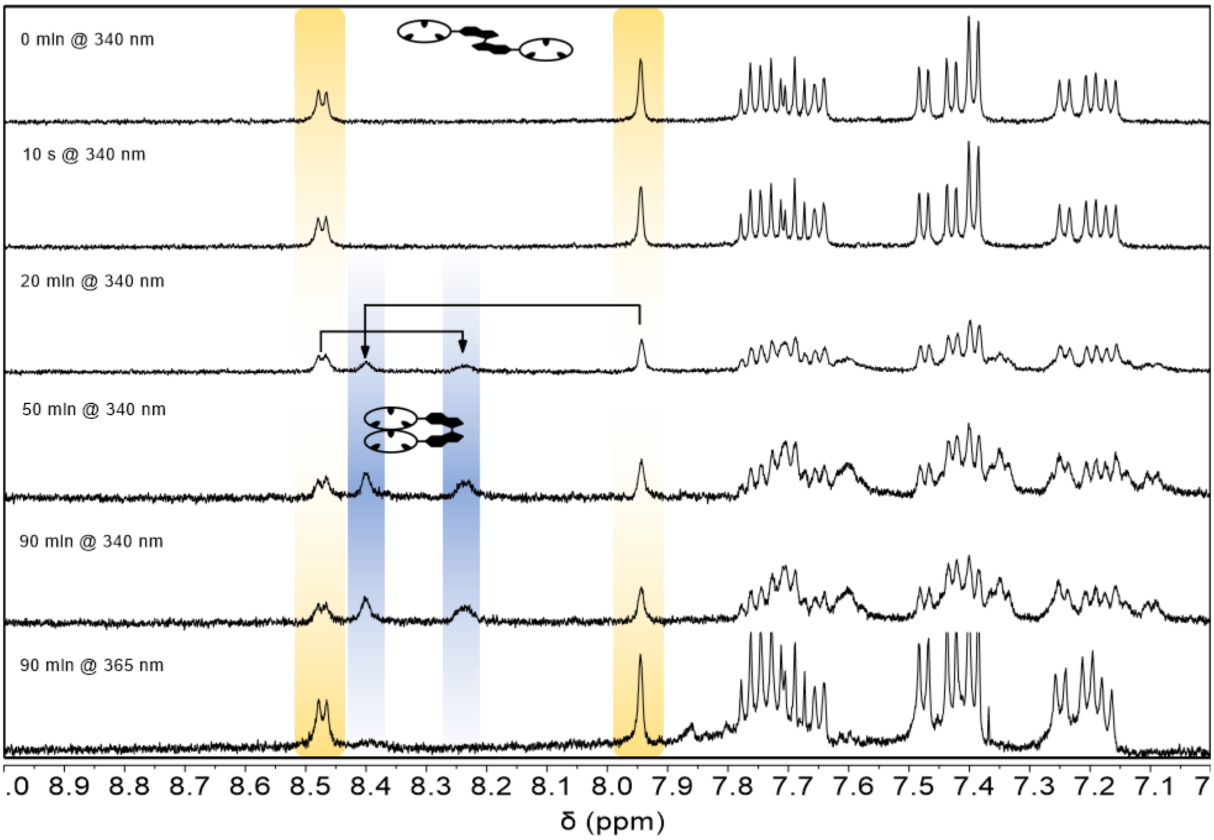

Figure S28. ¹H NMR spectra (500 MHz, 293 K) of the aromatic region of (*E*)-bis**CP** in 0.5 vol% H_2O/DMSO-d_6 (0.5 mM) and spectral changes after irradiation with 340 nm light ($PSS_{340}^{E/Z}$ ratio of 50:50) followed by irradiation with 365 nm ($PSS_{365}^{E/Z}$ > 95:5) at 20 °C.

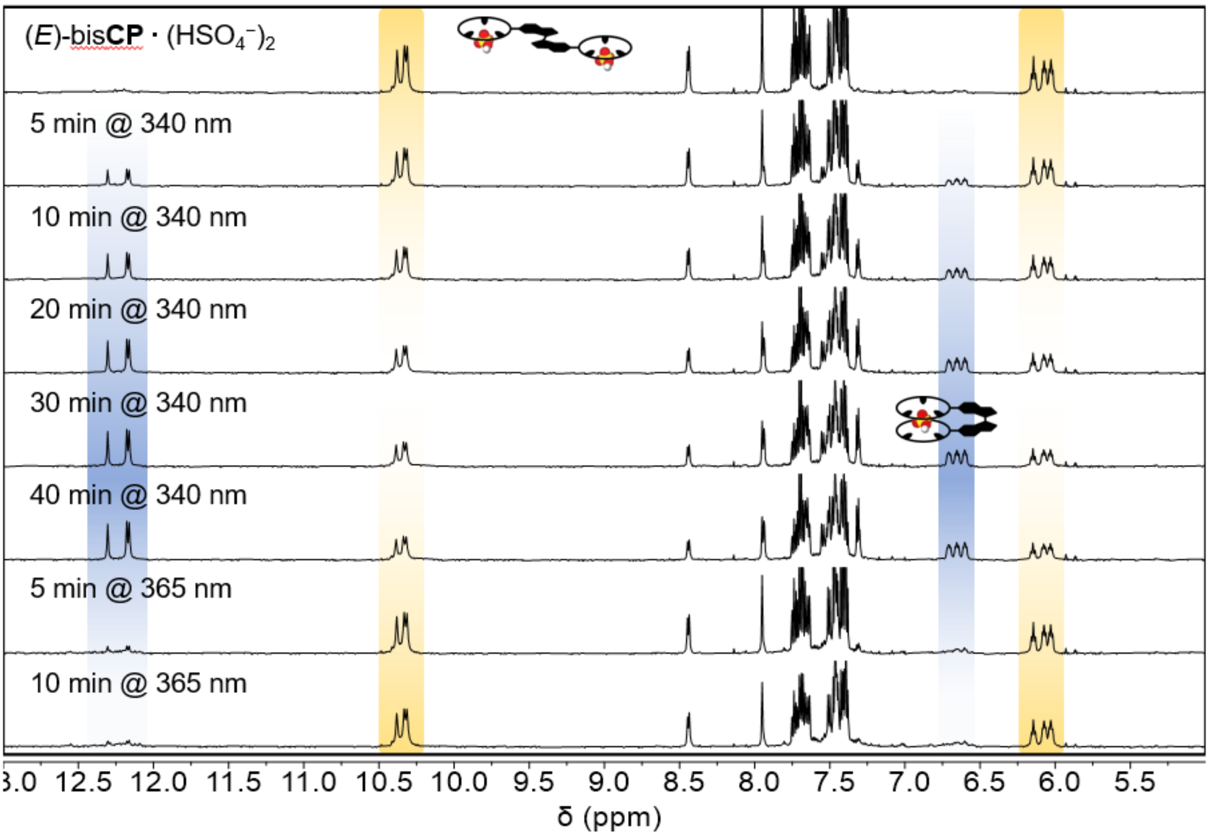

Figure S29. ^{1}H NMR spectra (500 MHz, 293 K) of the aromatic region of (*E*)-bis**CP** (0.5 mM) in the presence of TBAHSO$_4$ (5 mM) and 0.4 equiv. of H_2SO_4 in 0.5 vol% H_2O/DMSO-d_6 (0.5 mM) and spectral changes after irradiation with 340 nm light ($PSS_{340}^{E/Z}$ ratio of 46:54, 40 min) followed by irradiation with 365 nm ($PSS_{365}^{E/Z}$ ratio of >95:5, 10 min) at 20 °C.

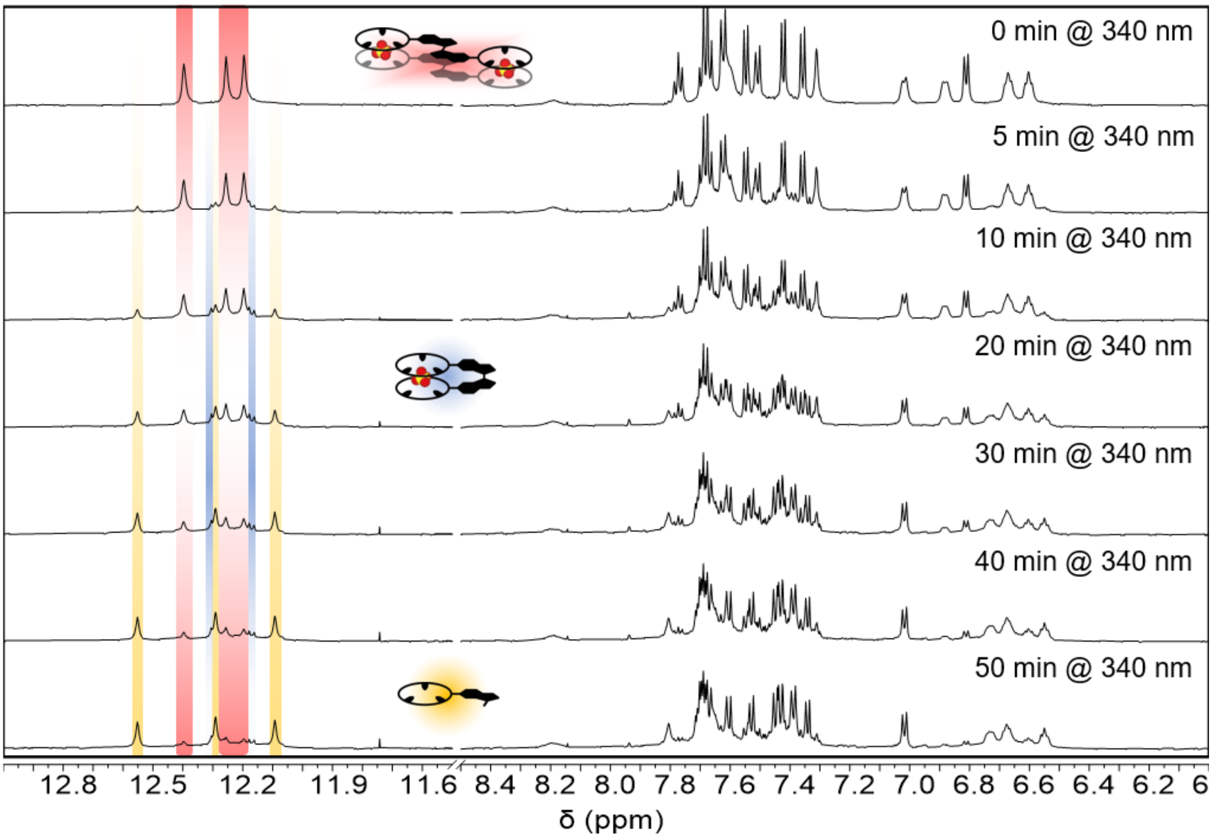

Figure S30. ^{1}H NMR spectra (500 MHz, 293 K) of [(*E*)-bis**CP**·SO_4^{2-}]$_2$ in 0.5 vol% H_2O/0.05 vol% DIPEA/DMSO-d_6 (0.5 mM) after irradiation with 340 nm light at 20 °C.

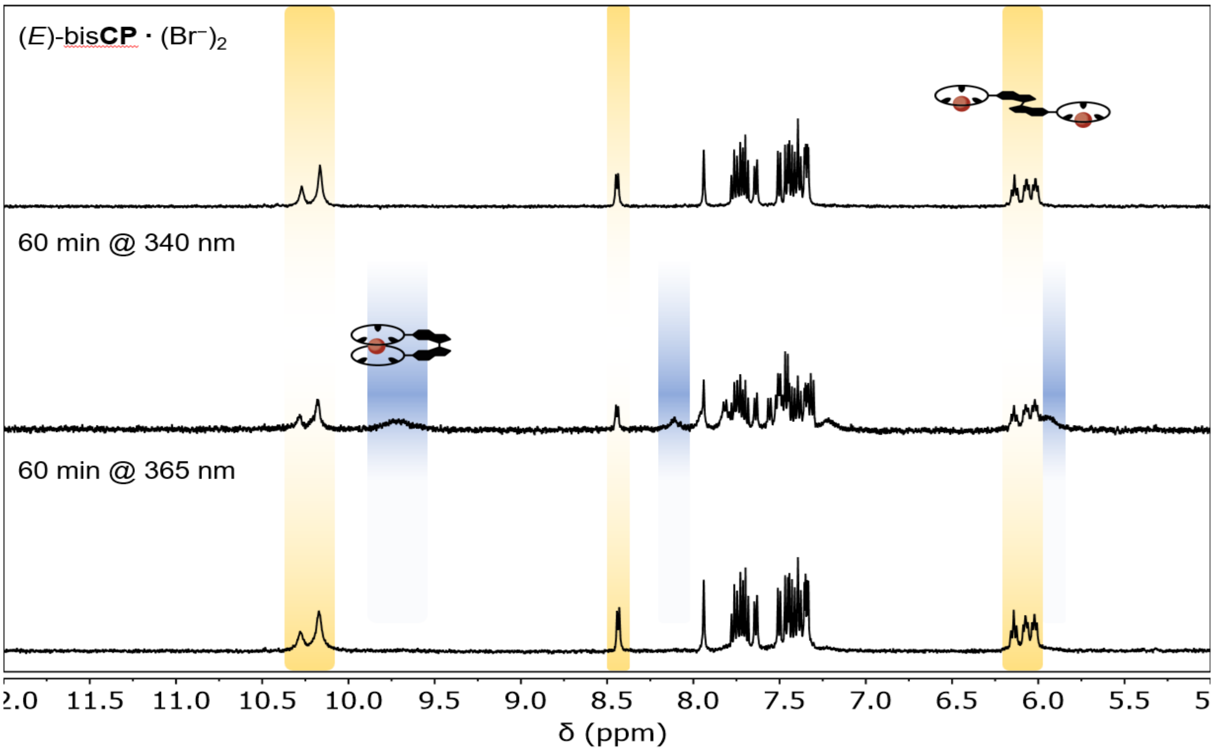

Figure S31. ^{1}H NMR spectra (500 MHz, 293 K) of the aromatic region of (*E*)-bis**CP** in the presence of TBABr (5 equiv.) in 0.5 vol% H_2O/DMSO-d_6 (0.5 mM) and spectral changes after irradiation with 340 nm light ($PSS_{340}^{E/Z}$ ratio of 63:37) followed by irradiation with 365 nm ($PSS_{365}^{E/Z}$ ratio of >95:5) at 20 °C.

5.2. Sample irradiation at elevated temperature

For the photoswitching studies at higher temperature, solutions of (*E*)- and (*Z*)-bis**CP** were prepared in 0.5 vol% H_2O/0.05 vol% DIPEA/DMSO-d_6 (0.5 mM), with the respective anion being added in 10-fold excess (5 mM). The NMR tube containing the sample was immersed sideways into an oil bath and irradiated from above (~10 cm distance), while being heated for a given amount of time. After regular time intervals, the sample was removed from the oil bath and a ^{1}H NMR spectrum was measured to track the configurational changes. Both species exhibited distinct chemical shifts for their respective NH proton signals. For [(*E*)-bis**CP**·SO_4^{2-}]$_2$, singlets appear at δ = 12.40, 12.26, and 12.20 ppm (red). For (*Z*)-bis**CP**·SO_4^{2-}, these signals shift to δ = 12.30, 12.18, and 12.16 ppm (blue), while for the decomposition product these signals appear at δ = 12.55, 12.29, and 12.09 ppm (yellow). For determination of the PSS ratios, the NH signals in the aromatic region of the (*E*)- and (*Z*)-isomer were integrated and compared.

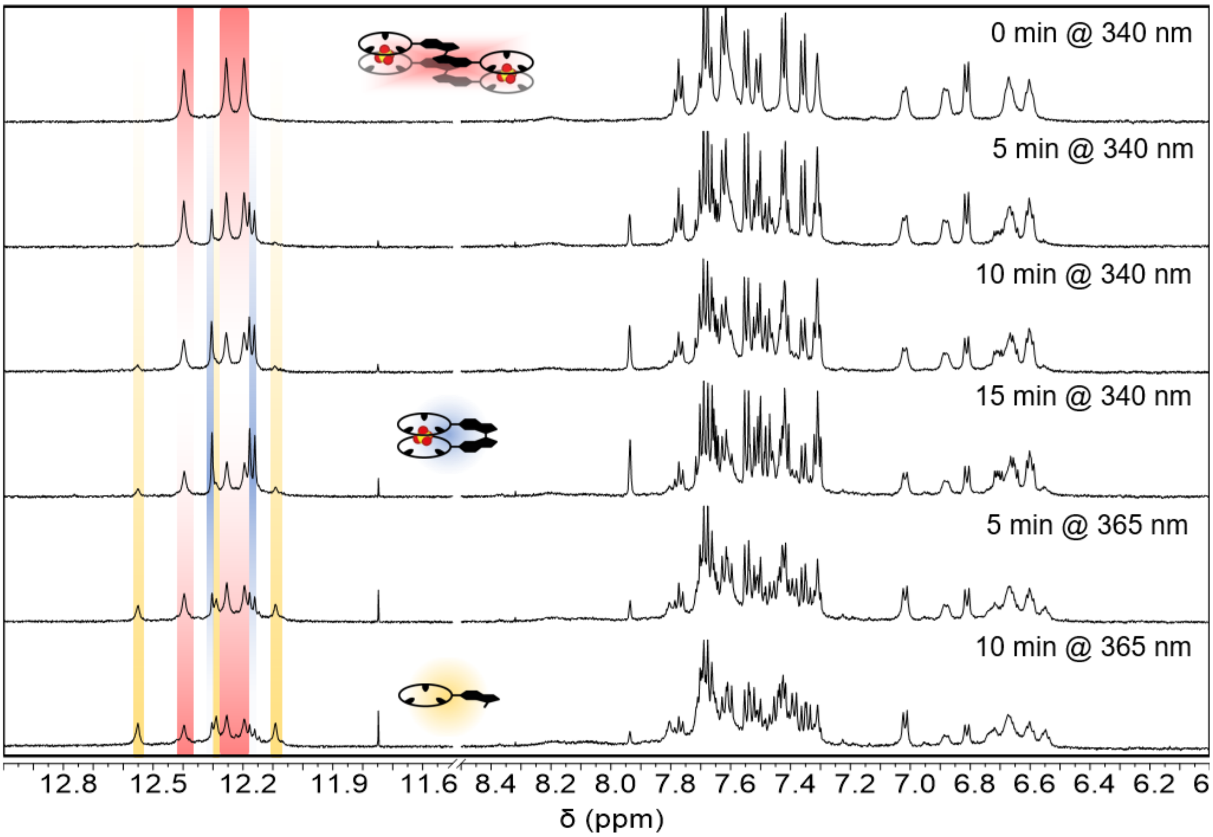

Figure S32. ^{1}H NMR spectra (500 MHz, 293 K) of [(*E*)-bis**CP**·SO_4^{2-}]$_2$ in 0.5 vol% H_2O/0.05 vol% DIPEA/DMSO-d_6 (0.5 mM) after irradiation with 340 nm ($PSS_{340}^{E/Z}$ ratio of 38:62, with 4% decomposition occurring) and 365 nm at 160 °C.

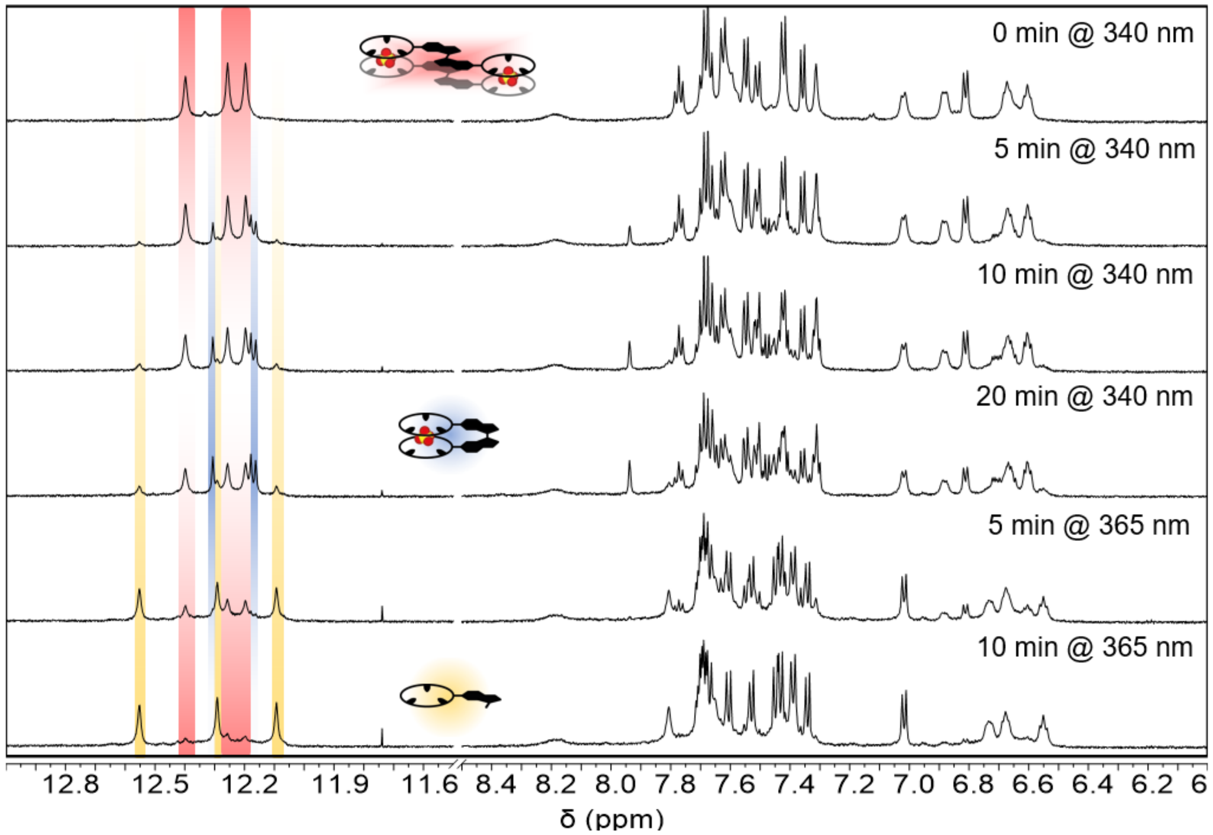

Figure S33. ^{1}H NMR spectra (500 MHz, 293 K) of [(*E*)-bis**CP**·SO_4^{2-}]$_2$ in 0.5 vol% H_2O/0.05 vol% DIPEA/DMSO-d_6 (0.5 mM) after irradiation with 340 nm ($PSS_{340}^{E/Z/decomp}$ ratio of 46:54, with 13% decomposition occurring) and 365 nm at 120 °C.

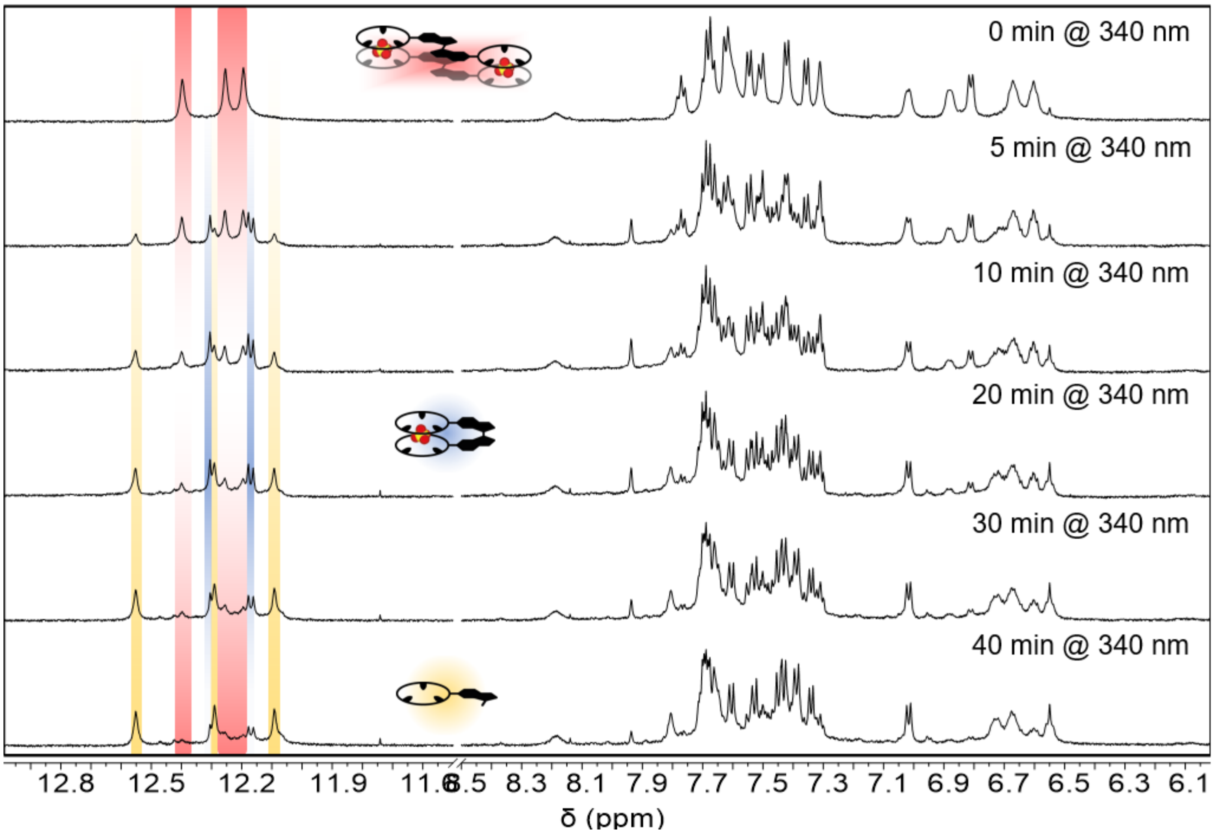

Figure S34. ^{1}H NMR spectra (500 MHz, 293 K) of [(*E*)-bis**CP**·SO_4^{2-}]$_2$ in 0.5 vol% H_2O/0.05 vol% DIPEA/DMSO-d_6 (0.5 mM) after irradiation with 340 nm ($PSS_{340}^{E/Z/decomp}$ ratio of 78:22, with 57% decomposition occurring) at 80 °C.

5.3. Identification of decomposition product

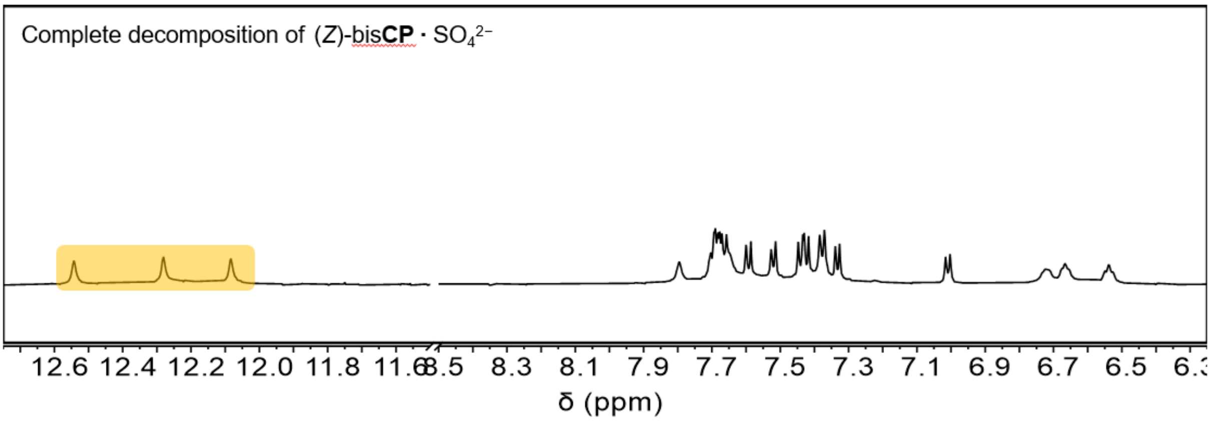

Figure S35. ^{1}H NMR spectra (500 MHz, 293 K) of the aromatic region of the decomposition product of (*Z*)-bis**CP**·SO_4^{2-} after prolonged irradiation with 365 nm light to reach full conversion, recorded in a mixture of 0.5 vol% H_2O/DMSO-d_6.

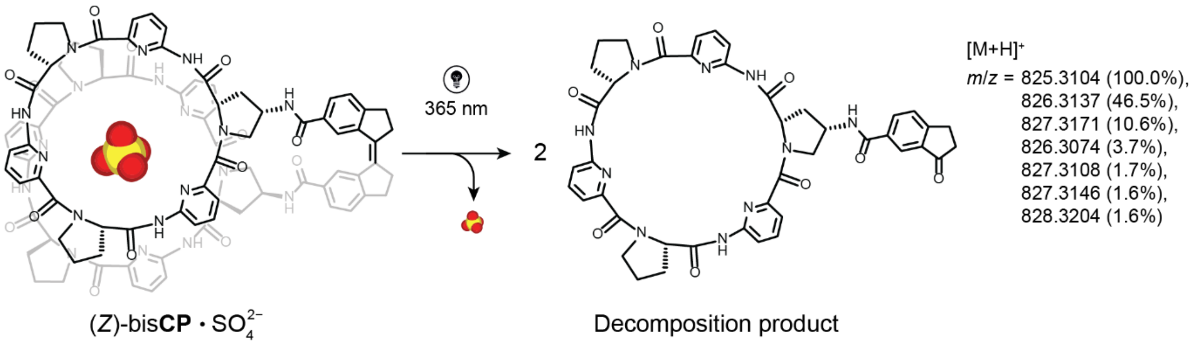

Figure S36. Decomposition of (*Z*)-bis**CP**·SO_4^{2-} after irradiation with 365 nm light to form a corresponding ketone and its *m*/*z* ratios for the singly charged species $[M+H]^+$.

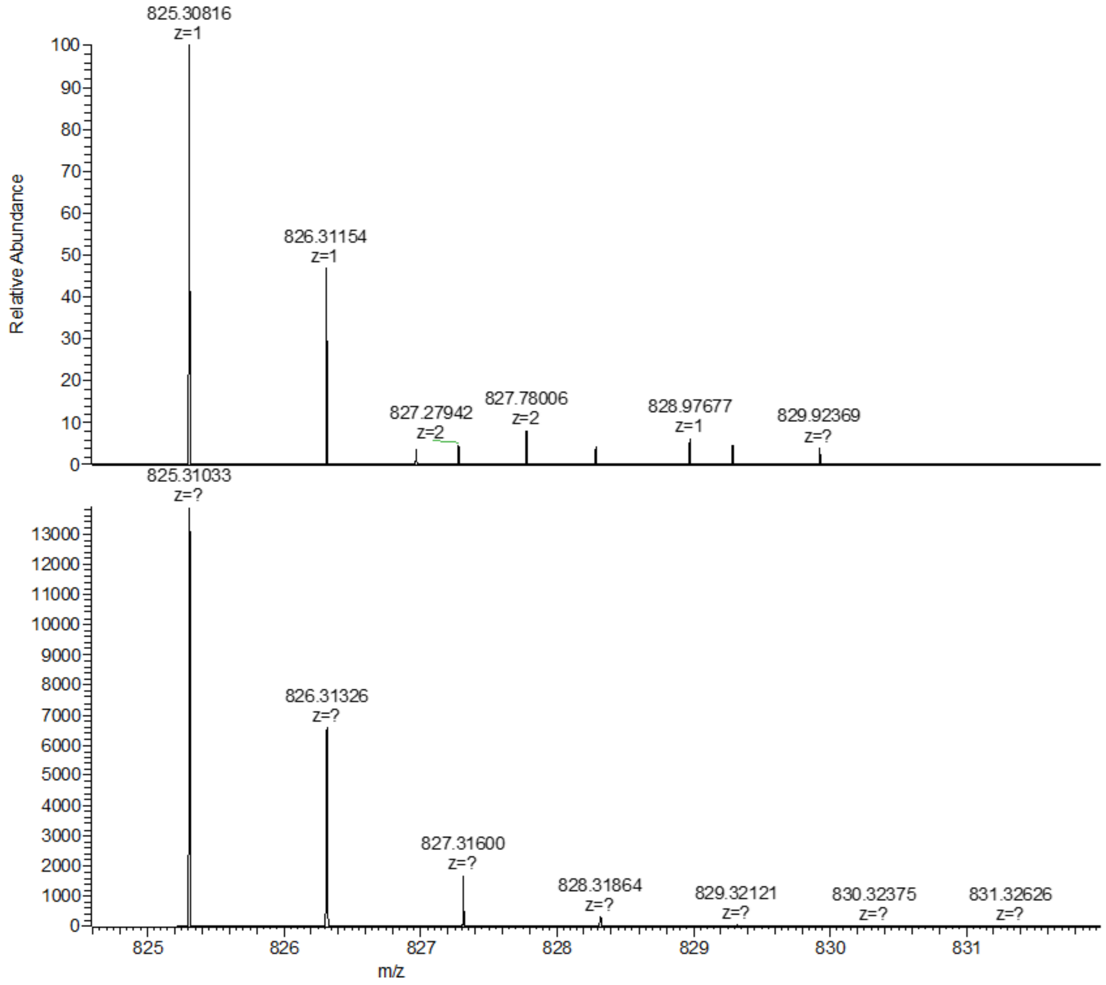

Figure S37. Recorded (top) and simulated mass spectrum (bottom) of the decomposition product of (*Z*)-bis**CP**·SO_4^{2-} after irradiation with 365 nm light proving the formation of the ketone, which is detected as singly charged species $[M+H]^+$.

6. ITC titrations

The ITC titrations were performed using a Microcal VP-ITC. Titrations with tetrabutylammonium hydrogensulfate (TBAHSO$_4$) were performed in DMSO containing 0.5 vol% of water. For the titrations with sulfate, TBAHSO$_4$ was used as salt and the above solvent mixture containing an additional 0.05 vol% of DIPEA. This solvent mixture was also used for the titrations with tetrabutylammonium bromide (TBABr).

The receptors and substrates were weighed by using an analytical precision balance, dissolved in known volumes of the corresponding solvent and loaded into the system for immediate analysis. Receptors and salts were dissolved in solvents from the same batch. The receptor and salt concentrations used are summarised in the following table.

Table S1: Concentrations of salts and receptors used in the ITC titrations.

Receptor	Salt	*c*(Receptor)/mM	*c*(Salt)/mM	Solvent
**CP**	TBAHSO$_4$	0.15	3.00	0.5 vol% H$_2$O/0.05 vol% DIPEA/DMSO
(*E*)-bis**CP**	TBABr	0.15	3.00	0.5 vol% H$_2$O/DMSO
	TBAHSO$_4$	0.15	3.00	0.5 vol% H$_2$O/DMSO
	TBAHSO$_4$	0.40	8.00	0.5 vol% H$_2$O/0.05 vol% DIPEA/DMSO
(*Z*)-bis**CP**	TBABr	0.15	3.00	0.5 vol% H$_2$O/DMSO
	TBAHSO$_4$	0.15	3.00	0.5 vol% H$_2$O/DMSO
	TBAHSO$_4$	0.40	8.00	0.5 vol% H$_2$O/0.05 vol% DIPEA/DMSO

An ITC experiment involved the titration of a solution of the salt into a solution of the receptor at 25 °C by using 34 injections of 3 µL at a rate of 0.25 µL s^{-1}, separated by an interval of 180 s, with the exception of the first injection, which was 2 µL. In the titration with TBABr, 4 µL of the salt solution were added during the 34 injections. The solutions were continuously stirred during the titrations at a rate of 611 rpm.

In the competitive titration of (*E*)-bis**CP** with sulfate, the solvent contained additional TBABr at a concentration of 15.0 mM.

To assess whether heats of dilution must be considered during data treatment, reference measurements were performed by titrating solutions of TBAHSO$_4$ in 0.5 vol% water/DMSO or 0.5 vol% water/0.05 vol% DIPEA/DMSO into the respective pure solvent. The heat effects observed in these measurements turned out to be very small (Figure S38), which is why heats of dilution were neglected when evaluating the binding isotherms.

Binding constants and enthalpies of binding were obtained by curve fitting of the titration data using the one-site binding model implemented in the Origin application provided by the

manufacturer of the ITC instrument. The competitive titration was evaluated using the competitive binding model implemented in Origin and the thermodynamic parameters independently determined for the TBABr complex of (*E*)-bis**CP**. The peak produced by the first injection and those of any obvious outliers were discarded prior to data processing. The thermograms and binding isotherms of selected measurements are shown in Figure S39-Figure S41.

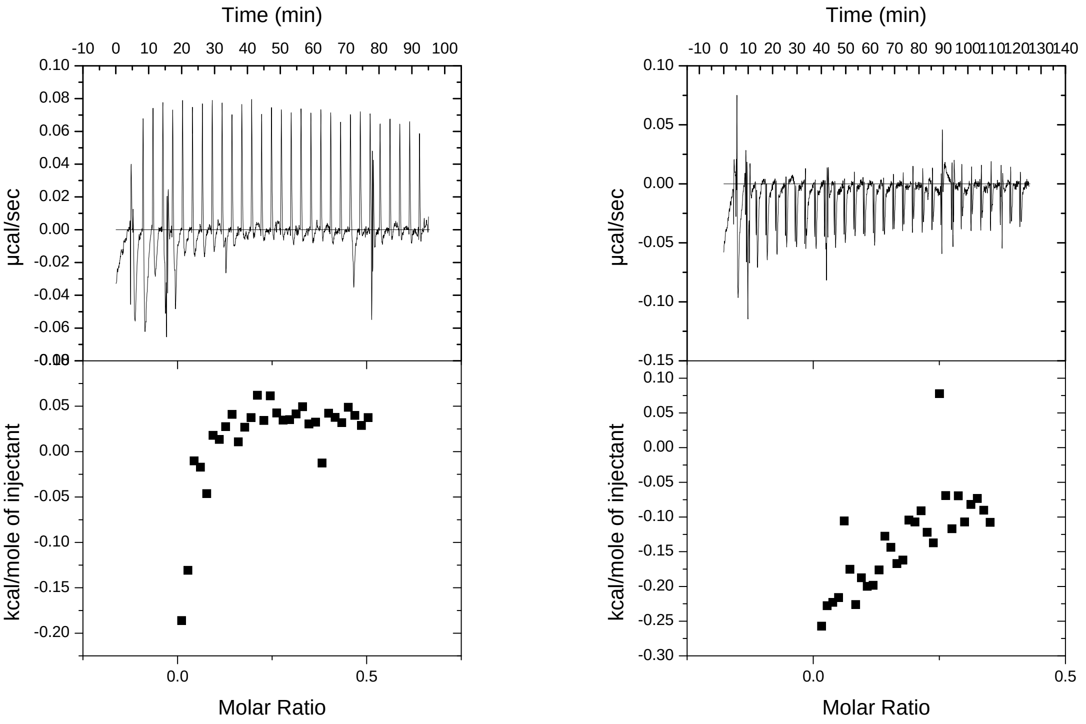

Figure S38. Titrations of $TBAHSO_4$ in 0.5 vol% H_2O/DMSO (left) or 0.5 vol% H_2O/0.05 vol% DIPEA/DMSO into the respective solvent mixture to estimate the heats of dilutions. The absolute heat effects are much smaller than those observed in the titrations, allowing the heat of dilution to be neglected.

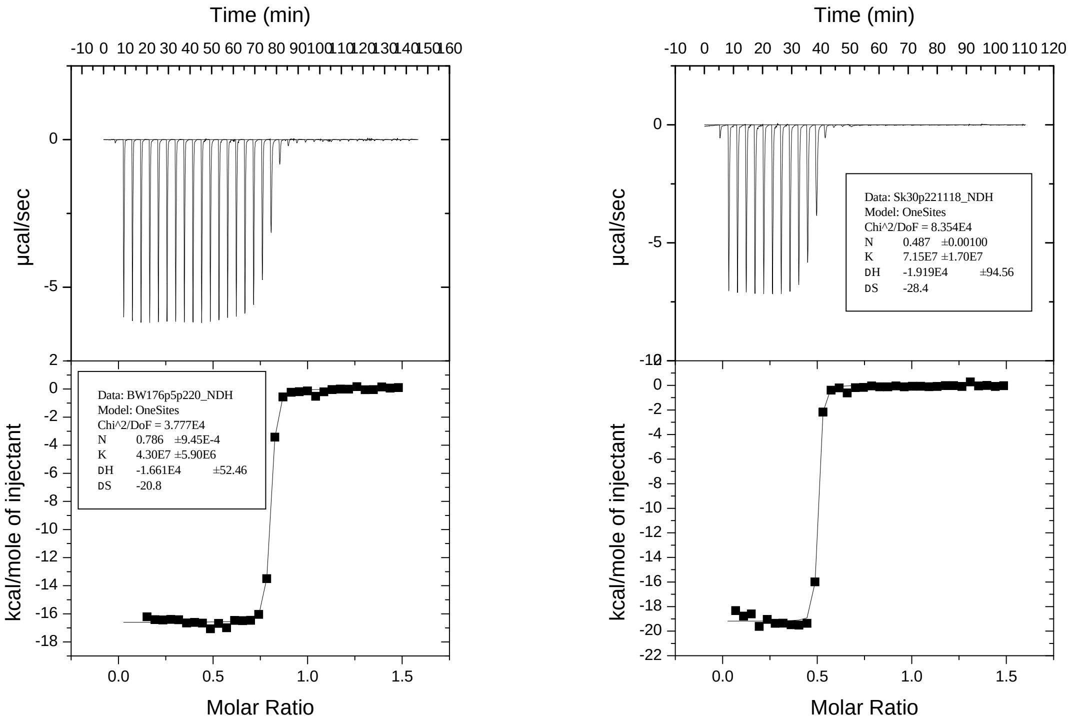

Figure S39. Titrations of (*E*)-bis**CP** (left) and model compound **CP** (right) with sulfate (TBAHSO4 in 0.5 vol% H_2O/0.05 vol% DIPEA/DMSO). The binding isotherms indicate strong binding with *N* value close to 1 and 0.5 suggesting the formation of [(*E*)-bis**CP**·SO_4^{2-}]$_2$ and [**CP**]$_2$·SO_4^{2-}, respectively. Both complexes form with positive cooperativity, since the corresponding isotherm features only a single step.

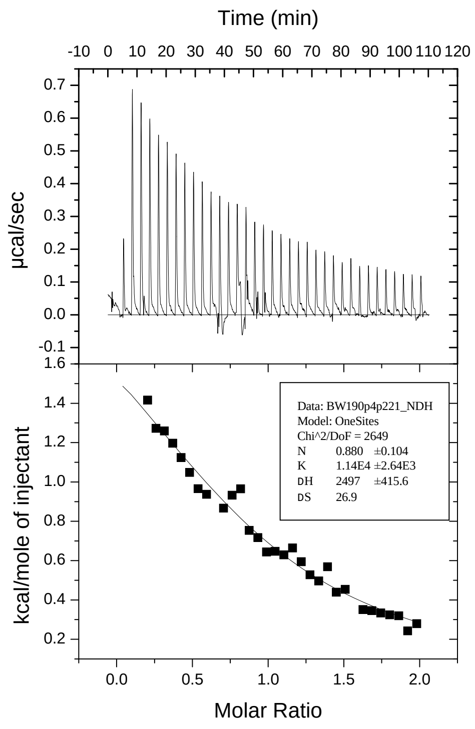

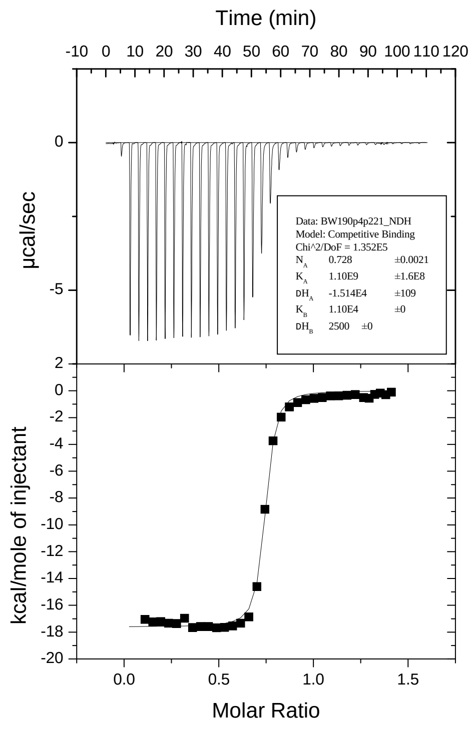

Figure S40. Titrations of (*Z*)-bis**CP** with bromide (TBABr in 0.5 vol% H_2O/0.05 vol% DIPEA/DMSO) (left). Competitive titration of (*Z*)-bis**CP** with sulfate ($TBAHSO_4$ in 0.5 vol% H_2O/0.05 vol% DIPEA/DMSO) in the presence of TBABr (15.0 mM) (right).

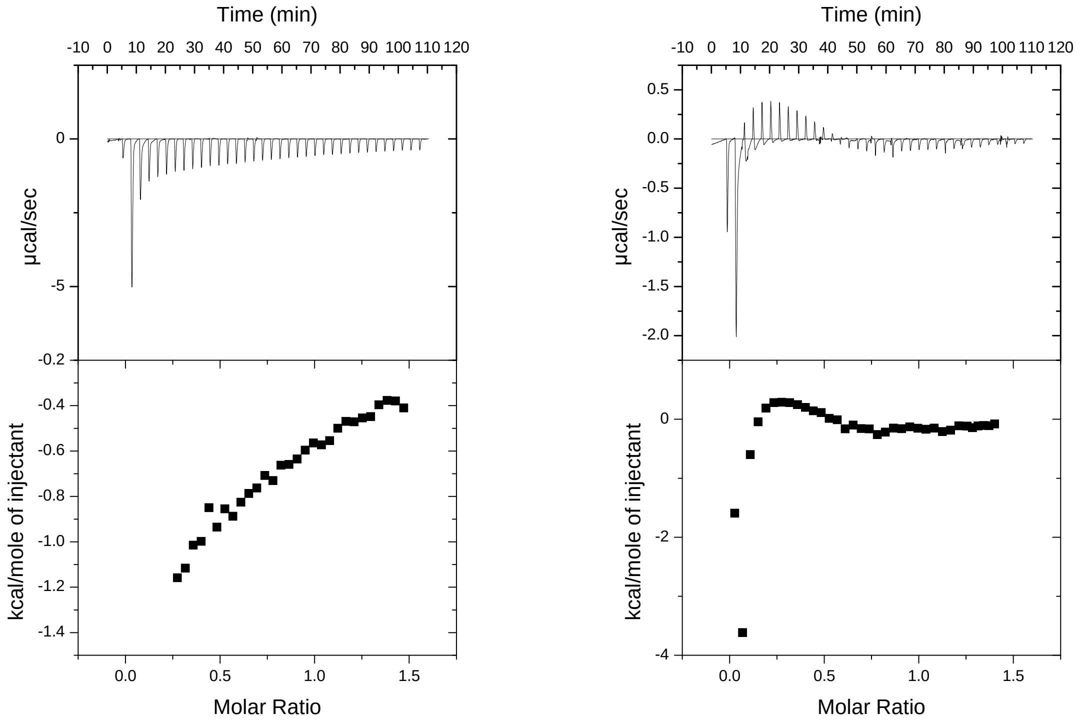

Figure S41. Titrations of (*E*)-bis**CP** (left) and (*Z*)-bis**CP** (right) with hydrogensulfate (TBAHSO$_4$ in 0.5 vol% H_2O/DMSO). Both titrations could not be fitted to a reasonable model. Binding is apparently weak.

7. DFT Calculations

For the calculations, the crystallographically determined coordinates of the sulfate complex of a previously described bis(cyclopeptide) were used.[6] The linking unit in this structure was replaced by the stiff-stilbene linker in (*Z*)-bis**CP** and the resulting structure was optimized in the gas phase using the MMFF force field implemented in Spartan 24 (Wavefunction Inc.). A DFT structure optimization was then performed using the B3LYP functional and the 6-31G* basis set.

For the calculation of the 2:2 complex of (*E*)-bis**CP**, the torsion angle at the double bond in the calculated sulfate complex of (*Z*)-bis**CP** was changed to 180°, two of these structures and two sulfate anions were pre-arranged in the expected manner, and the MMFF and subsequent DFT calculation were repeated.

Note that the resulting structures should only serve to illustrate the structural complementarity of the bis(cyclopeptides) in their anion complex and the structural effects of the stiff-stilbene linkers. The molecules are flexible, and many structures are likely to exist with similar subunit arrangements but slightly different conformations of the cyclopeptide rings and linkers and orientations of the anions.

The coordinates of both complexes have been made available as separate files.

8. References

1 S. Akbulatov, Y. Tian and R. Boulatov, *J. Am. Chem. Soc.* 2012, **134**, 7620-7623.

2 D. Villarón, M. A. Siegler and S. J. Wezenberg, *Chem. Sci.*, 2021, **12**, 3188-3193.

3 T. Fiehn, R. Goddard, R. W. Seidel and S. Kubik, *Chem. Eur. J.* 2010, ***16,*** 7241-7255.

4 P. Gans, A. Sabatini and A. Vacca, *Talanta*, 1996, ***43***, 1739-1753.

5 S. J. Wezenberg and B. L. Feringa, *Org. Lett.* 2017, **19**, 324-327.

6 Z. Rodriguez-Docampo, S. I. Pascu, S. Kubik and S. Otto, *J. Am. Chem. Soc.*, 2006, **128**, 11206-11210.
